# qSOFA combined with suPAR for early risk detection and guidance of antibiotic treatment in the emergency department: a randomized controlled trial

**DOI:** 10.1186/s13054-024-04825-2

**Published:** 2024-02-06

**Authors:** Maria Evangelia Adami, Antigone Kotsaki, Nikolaos Antonakos, Efthymia Giannitsioti, Stamatios Chalvatzis, Maria Saridaki, Christina Avgoustou, Karolina Akinosoglou, Konstantina Dakou, Georgia Damoraki, Konstantina Katrini, Panagiotis Koufargyris, Vasileios Lekakis, Antonia Panagaki, Asimina Safarika, Jesper Eugen-Olsen, Evangelos J. Giamarellos-Bourboulis

**Affiliations:** 1 Fourth Department of Internal Medicine, Medical School, National and Kapodistrian University of Athens, ATTIKON University General Hospital, 1 Rimini Str, 124 62 Athens, Greece; 2 Department of Internal Medicine, University of Patras, Rion, Greece; 3 Hellenic Institute for the Study of Sepsis, Athens, Greece; 4 Department of Clinical Research, Copenhagen University Hospital Amager and Hvidovre, Hvidovre, Denmark

**Keywords:** Meropenem, Sepsis, Risk, suPAR

## Abstract

**Background:**

Sepsis guidelines suggest immediate start of resuscitation for patients with quick Sequential Organ Failure Assessment (qSOFA) 2 or 3. However, the interpretation of qSOFA 1 remains controversial. We investigated whether measurements of soluble urokinase plasminogen activator receptor (suPAR) may improve risk detection when qSOFA is 1.

**Methods:**

The study had two parts. At the first part, the combination of suPAR with qSOFA was analyzed in a prospective cohort for early risk detection. At the second part, the double-blind, randomized controlled trial (RCT) SUPERIOR evaluated the efficacy of the suPAR-guided medical intervention. SUPERIOR took place between November 2018 and December 2020. Multivariate stepwise Cox regression was used for the prospective cohort, while univariate and multivariate logistic regression was used for the RCT. Consecutive admissions at the emergency department (ED) with suspected infection, qSOFA 1 and suPAR ≥ 12 ng/mL were allocated to single infusion of placebo or meropenem. The primary endpoint was early deterioration, defined as at least one-point increase of admission Sequential Organ Failure Assessment (SOFA) score the first 24 h.

**Results:**

Most of the mortality risk was for patients with qSOFA 2 and 3. Taking the hazard ratio (HR) for death of patients with qSOFA = 1 and suPAR < 12 ng/mL as reference, the HR of qSOFA = 1 and suPAR ≥ 12 ng/mL for 28-day mortality was 2.98 (95% CI 2.11–3.96). The prospective RCT was prematurely ended due to pandemia-related ED re-allocations, with 91 patients enrolled: 47 in the placebo and 44 in the meropenem arm. The primary endpoint was met in 40.4% (*n* = 19) and 15.9% (*n* = 7), respectively (difference 24.5% [5.9–40.8]; odds ratio 0.14 [0.04–0.50]). One post hoc analysis showed significant median changes of SOFA score after 72 and 96 h equal to 0 and − 1, respectively.

**Conclusions:**

Combining qSOFA 1 with the biomarker suPAR improves its prognostic performance for unfavorable outcome and can help decision for earlier treatment.

*Trial registration* EU Clinical Trials Register (EudraCT, 2018-001008-13) and Clinical-Trials.gov (NCT03717350). Registered 24 October 2018.

**Supplementary Information:**

The online version contains supplementary material available at 10.1186/s13054-024-04825-2.

## Introduction

Early recognition and start of antibiotics are the cornerstone of sepsis management [1]. Since early recognition often fails, warning scores have been introduced to facilitate early recognition [2–5]. qSOFA (quick Sequential Organ Failure Assessment Score) is one simplistic approach which integrates mental confusion with increase in the respiratory rate and hypotension; patients with suspicion of infection and at least two of these clinical signs are at nearly threefold greater risk for death after 28 days [6]. Early sepsis resuscitation is warranted for patients with 2 or 3 qSOFA points. However, there is ambivalence how to manage patients with one qSOFA point [7, 8].

Previous studies of our group showed that blood concentrations of the biomarker suPAR (soluble urokinase plasminogen activator receptor) 12 ng/mL or more are an independent predictor of death the first 28 days for patients with infection [9]. suPAR is the soluble form of the membrane-bound receptor uPAR on myeloid cells cleaved after an inflammatory stimulus which promotes chemotaxis and cell migration [10–12]. We hypothesized that suPAR may improve the early detection of sepsis in patients with one sign of qSOFA.

Current guidelines of the Surviving Sepsis Campaign do not suggest in favor of qSOFA for the early detection of sepsis [13]. The analysis of data coming from the Hellenic Sepsis Study Group (HSSG) indicates significant risk for death among infections with one qSOFA sign upon admission [7]. This stimulated us to follow an investigational approach including two parts. In the first part, the risk of death was defined among patients with one qSOFA sign and increased suPAR in a retrospective analysis of one large-scale prospective study. In the second part, we designed and conducted the randomized controlled trial with the acronym SUPERIOR (SUPar-guided doublE-blind randomized controlled trial of Initiation Of antibiotics foR presumed infection at the emergency department). The aim of the SUPERIOR trial was to investigate whether early antibiotic treatment for patients with one qSOFA sign and increased suPAR may impact on patients’ outcomes.

## Methods

### Part 1: prospective HSSG registry

#### Study design and data source

The HSSG is running one prospective registry of clinical data and biomaterials collected from patients admitted to 39 departments in Greece which are departments of internal medicine, surgery and intensive care units (www.sepsis.gr) (see Additional file 1: Table S1). The samples used for this study were collected between May 2006 and December 2016. Since at that study period, the Sepsis-3 definitions were not yet introduced, patients were re-classified retrospectively for qSOFA signs. The study protocol was approved by the Ethics Committees of the participating hospitals, and patients were enrolled after written informed consent provided by themselves or their first-degree relatives (see Additional file 1: Table S1).

#### Study population

Participants were aged 18 years or more, with acute pyelonephritis or lung infection or intra-abdominal infection, and had at least two signs of the systemic inflammatory response syndrome (SIRS). Main exclusion criteria were known infection by the human immunodeficiency virus and neutropenia (see Additional file 2).

#### Variables, exposures and endpoints

Blood was sampled the first 24 h from onset of SIRS. The following information were captured: severity scores, comorbidities, microbiology, administered antibiotics and 28-day outcome. suPAR was measured in duplicate in serum using a CE/IVD enzyme immunosorbent assay (suPARnostic ELISA, ViroGates, Denmark). The lower limit of detection was 0.4 ng/mL.

The aim of the analysis was to investigate whether for patients with one qSOFA sign, suPAR 12 ng/mL or more may be an independent predictor of 28-day outcome.

### Statistical analysis

Demographics were expressed as frequencies and 95% confidence intervals (CI) for qualitative data, and as means (SD) or median (quartiles) for quantitative variables with normal or non-normal distribution. Analysis was done by multivariate stepwise Cox regression analysis; hazard ratios (HR) and 95% CI for the independent factors associated with 28-day mortality were defined. The serum suPAR cutoff level with the best trade-off between sensitivity and specificity to predict 28-day mortality among patients with qSOFA equal to 1 was calculated after design of the receiver operator characteristics (ROC) curve using the Youden index. Any value of (two-sided) p less than 0.05 was considered significant. Analysis was performed using IBM SPSS Statistics v23.

### Part 2: the SUPERIOR study

#### Study design and data source

SUPERIOR is a prospective, double-blind RCT conducted in two tertiary University hospitals in Greece. The study protocol was approved by the Ethics Committees of the participating hospitals (approval 276/03–05-2018 by ATTIKON University General Hospital; approval 1060/05–12-2018 by Rion University General Hospital), by the National Ethics Committee of Greece (106/24–07-2018) and by the National Organization for Medicines of Greece (approval 141/ 21–05-2020). The study is prospectively registered at the EU registry database (2018–001008-13) and at Clinicaltrials.gov (NCT03717350). Written informed consent was provided by the patients or legal representatives.

#### Study population

Included patients were male or female adults admitted to the emergency department (ED) with clinical suspicion of infection, one qSOFA sign and suPAR in the blood 12 ng/mL or more. Main exclusion criteria were: patients scoring 0, 2 or 3 points of qSOFA; pregnancy or lactation, organ transplantation, full-blown sepsis requiring immediate resuscitation as defined by the treating physicians or a decision not to resuscitate (DNR).

Patients meeting all inclusion criteria and none of the exclusion criteria were 1:1 randomized to either one intravenous dose of placebo or 2 g meropenem. Stratified randomization was performed using a computer-generated sequence. Study drug was prepared by an unblinded pharmacist who had access to the electronic study system using separate username and password. The prepared drugs were similar in appearance. The study drug was prepared in a final volume of 100 mL in 0.9% sodium saline and it was infused intravenously within 15 min. The drug was administered by a blinded study nurse.

#### Variables, exposures and endpoints

suPAR was measured in human EDTA plasma using the rapid suPARnostic® Quick Triage (ViroGates, Denmark), a lateral flow immunoassay which provides results in 20 min. The assay provides a quantitative suPAR measurement in ng/mL, ranging from 2 to 15 ng/mL. If samples were above 15 ng/mL, the sample was diluted 1:1 with dilution buffer and rerun.

The daily clinical assessment including clinical symptoms, laboratory tests, vital-sign assessment tools, type of infection, resolution of infection and survival was performed by blind investigators until hospital discharge or death. The type of infection was classified using pre-defined criteria (see Additional file 1: Table S2). All discharged patients were followed up by telephone calls for clinical condition and health state until day 90. New infections or hospitalizations were recorded. Treatment-emergent adverse events (TEAEs) were recorded and classified into serious and non-serious. Patients who failed study enrollment because of suPAR less than 12 ng/mL were followed up until day 28 for survival.

The primary study endpoint was the early worsening of the patient. This was defined as any increase of total admission SOFA score by at least one point the first 24 h. The key secondary endpoint was the validation of the predictive performance of increased suPAR for 28-day mortality. For this purpose, 28-day mortality was compared between patients failing screening because of suPAR less than 12 ng/mL and patients enrolled in the study and allocated to treatment with placebo.

Other secondary endpoints were the increase of admission total SOFA score by at least two points the first 24 h; mortality by days 7, 28, 60 and 90; time to infection resolution; change of initial antibiotics; duration of hospital stay; and incidence of new infections the first 60 days.

### Statistical analysis

The study was powered for the primary endpoint. With the assumptions of 80% power at the 10% level of significance and that the primary endpoint would be achieved in 30% of patients allocated to the placebo arm and 15% of patients allocated to the meropenem arm, 110 patients were needed in each group. The study was analyzed for the intention-to-treat (ITT) population. Categorical variables were expressed as frequencies and 95% CIs. Continuous variables were expressed as mean and standard deviation (SD) or median and interquartile range (IQR), as appropriate. Comparisons for categorical variables were made using the Chi-square test or Fischer’s exact test. For continuous variables, comparisons were made with the nonparametric Mann–Whitney U test or Student’s t test. Univariate and multivariate logistic regression models were performed to identify variables and factors associated with the primary outcome. Results were expressed as odds ratio (OR) and 95% confidence intervals (CI). Hosmer and Lemeshow’s test was used as goodness of fit for multivariable model. Covariates included in the multivariate model were baseline characteristics of study participants which met at least one of two conditions: (a) the *p* value of comparison between patients achieving or not the primary endpoint was less than 0.100; or (b) the *p* value of comparison between the two groups was less than 0.100. As the change of SOFA score after 72 h and 96 h is suggested important surrogate for the disease course by others [14, 15], a post hoc analysis was done. In that analysis, the delta SOFA at 72 and 96 h was calculated as the difference between the SOFA score of days 4 and 5 from the SOFA score on admission. Comparisons between groups were done by the Mann–Whitney U test. Any value of P less than 0.05 was considered statistically significant. Analysis was conducted using IBM SPSS Statistics v26.

## Results

### Part 1: prospective HSSG registry

A total of 2,377 patients from the HSSG registry were analyzed (see Additional file 3: Fig. S1). Patients were divided into four groups: group A including 590 patients with 0 qSOFA signs; group B including 615 patients with one qSOFA sign and suPAR less than 12 ng/mL; group C including 290 patients with one qSOFA sign and suPAR ≥ 12 ng/mL; and group D including 882 patients with 2 or 3 qSOFA signs (Additional file 1: Table S2). ROC analysis among patients with qSOFA equal to 1, defined suPAR 12 ng/mL or more as the best trade-off between sensitivity and specificity. This cutoff had 88.5% negative predictive value to exclude the risk for death after 28 days (see Additional file 4: Fig. S2).

**Fig. 1:**
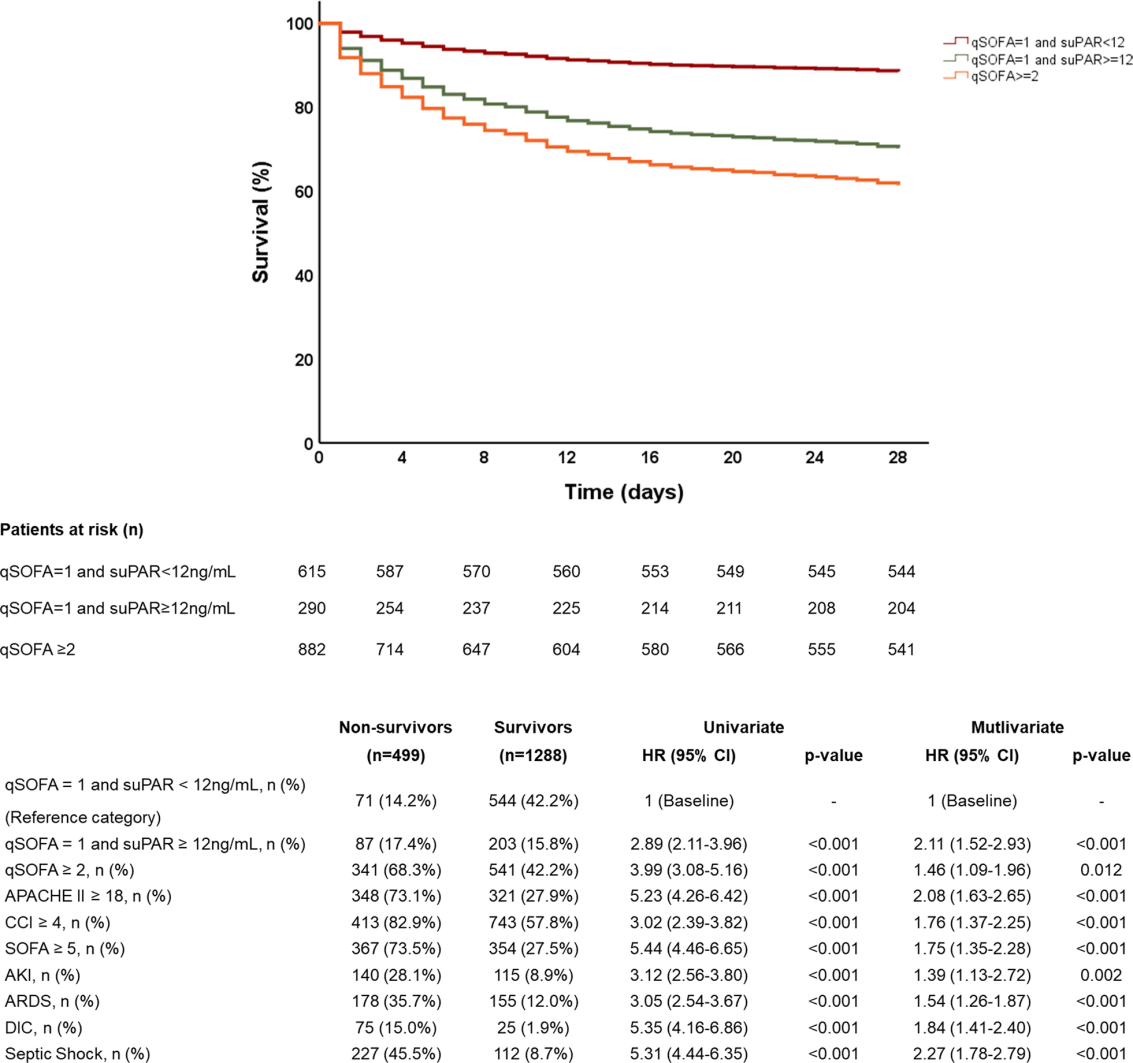
Improvement of risk prediction for unfavorable outcome by the qSOFA and suPAR combination. Survival curves provide the analysis of 1787 patients enrolled in the HSSG prospective cohort stratified into three strata of severity by qSOFA score and serum suPAR. The table provides the stepwise Cox regression analysis of survival for each stratum of severity. Cutoffs of APACHE II score, CCI and SOFA score were defined after ROC analyses. *CI* Confidence interval, *AKI* acute kidney injury, *ARDS* acute respiratory distress syndrome, *APACHE* acute physiology and chronic health evaluation, *DIC* disseminated intravascular coagulation, *HSSG* Hellenic Sepsis Study Group, *HR* hazard ratio, *ROC* receiver operator characteristics curve, *SOFA* Sequential Organ Failure Assessment Score, *suPAR* soluble urokinase plasminogen activator receptor

The 28-day mortalities were 7.5% (95% CI 5–10%) for group A, 11.5% (95% CI 9–14%) for group B, 30% (95% CI 25–35%) for group C and 38.7% (95% CI 35–42%) for group D. In the forward stepwise Cox regression analysis patients with one qSOFA point and suPAR less than 12 ng/mL were entered as the reference point. The presence of both one qSOFA point and increased suPAR increased significantly the risk of death (HR: 2.98; 95% CI 2.11–3.96) and even provided similar mortality risk as qSOFA 2 or more (HR: 3.99; 95% CI 3.08–5.16) (Fig. 1A,B). An additive risk for death was found after combining suPAR with any qSOFA point (Additional file 1: Table S3).

### Part 2: the SUPERIOR study

**Fig. 2:**
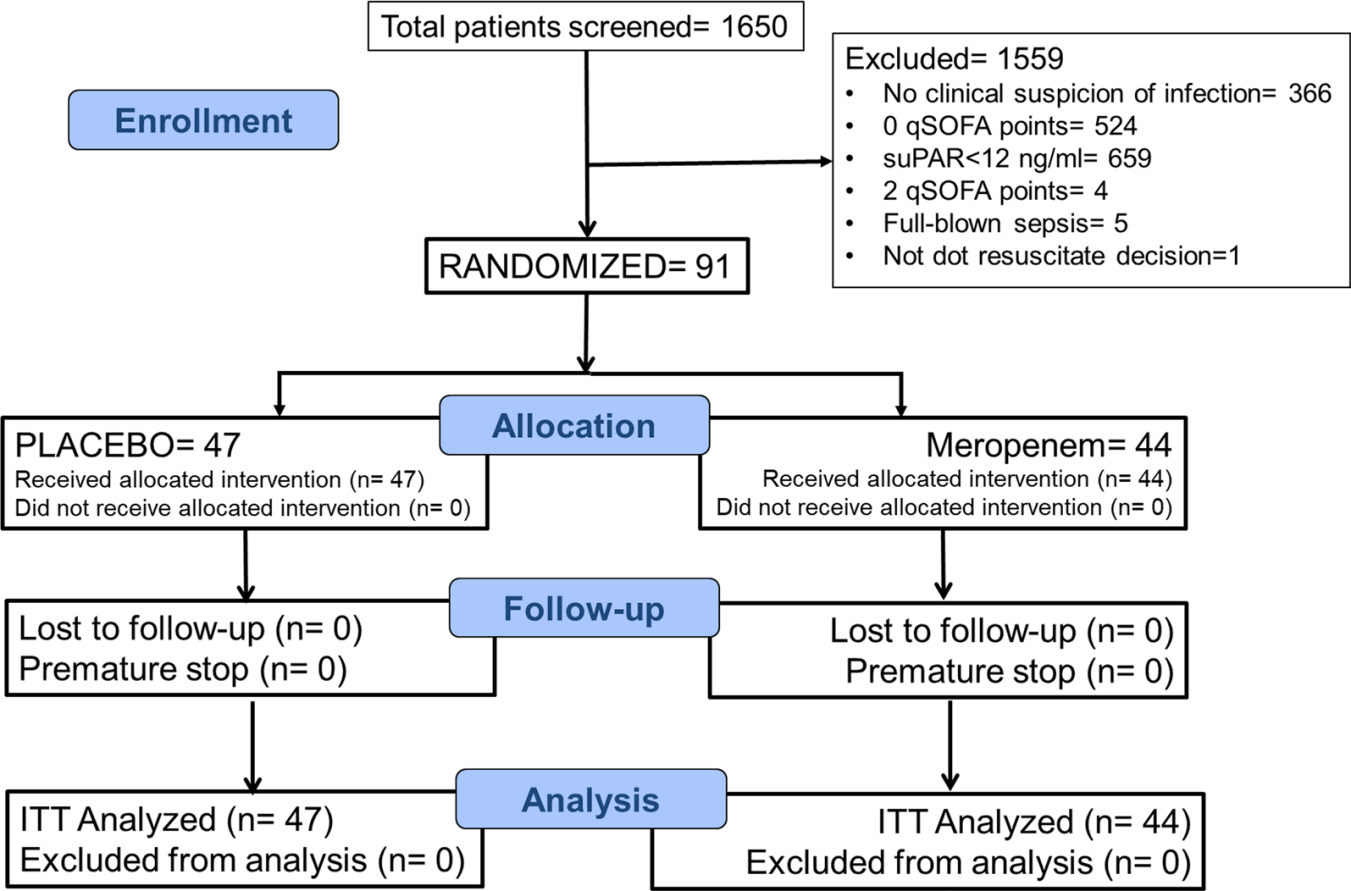
Flowchart of the SUPERIOR trial. *ΙΤΤ* Intent-to-treat, *n* number of patients, *qSOFA* Quick Sequential Organ Failure Assessment Score, *suPAR* soluble urokinase plasminogen activator receptor

The first patient was enrolled on November 12, 2018. The study was stopped prematurely by the Sponsor in December 2020. The reason of premature stop was the COVID-19 pandemic during which the function of the ED taking care only of patients infected by SARS-CoV-2 did not logistically allow the study to run. The last visit of the last patient was completed on January 13, 2021. During the study period, 91 patients were enrolled: 47 allocated to the placebo group and 44 to the meropenem group (Fig. 2). Eighty-four patients were enrolled before the start of the pandemic. If the study could run in a similar enrollment rate, the pre-calculated number of patients would have been enrolled in due course. However, the enrollment of only seven patients after the start of the pandemic made evident that the study could not continue at the desired rate of enrollment and led to the decision of premature stop. Baseline characteristics were similar in both groups (Table 1 and Additional file 1: Table S4). The median time from blood drawing until suPAR result was 40 min in both groups; and from blood drawing until start of the study drug was 50 min in both groups.

**Table 1:** Baseline characteristics of patients enrolled in the SUPERIOR trial before randomization

	Placebo (*n* = 47)	Meropenem (*n* = 44)	*p* value
Male gender, *n* (%)	15 (31.9)	23 (52.3)	0.058
Age, years, mean (SD)	73.2 (16.4)	73.8 (14.4)	0.858
CCI, mean (SD)	5.49 (2.84)	5.89 (3.01)	0.519
Comorbidities, *n* (%)			
Type 2 diabetes mellitus	14 (29.8)	15 (34.1)	0.822
COPD	9 (19.1)	8 (18.2)	1.00
Chronic heart failure	10 (21.3)	13 (29.5)	0.472
Chronic renal disease	17 (36.2)	15 (34.1)	1.00
Coronary heart disease	5 (10.6)	12 (27.3)	0.059
Atrial fibrillation	14 (29.8)	13 (29.5)	1.00
Parkinson’s disease	4 (8.5)	0 (0)	0.118
Ischemic stroke	6 (12.8)	3 (7)	0.489
Baseline MH SOFA score, mean (SD)	1.51 (1.73)	1.34 (1.38)	0.608
Admission ED SOFA score, mean (SD)	3.04 (2.44)	3.43 (1.78)	0.390
Admission APACHE II score, mean (SD)	12.78 (5.47)	14.18 (5.32)	0.223
White blood cell count, (/mm^3^), mean (SD)	10,387.2 (4313.4)	12,272.1 (7731.2)	0.152
Platelet cell count, (/mm^3^), mean (SD)	249,255.3 (116,691.8)	235,418.6 (92,547.8)	0.537
qSOFA signs, *n* (%)			
Respiratory rate ≥ 22/min	40 (85.1)	39 (88.6)	0.760
Systolic blood pressure < 100 mmHg	5 (10.6)	4 (9.1)	1.00
Altered mental status	2 (4.3)	1 (2.3)	1.00
Creatinine, mg/dl, mean (SD)	2.05 (2.19)	3.04 (6.04)	0.301
AST, U/l, mean (SD)	47.9 (47.7)	46.4 (43.9)	0.883
ALT, U/l, mean (SD)	34.2 (49.4)	40.7 (36.6)	0.487
Total bilirubin, mean (SD)	1.04 (1.53)	1.27 (1.84)	0.524
Final diagnosis, *n* (%)			
Community-acquired pneumonia	13 (27.6)	12 (27.3)	1.00
Health care associated pneumonia	11 (23.4)	11 (25.0)	1.00
Acute pyelonephritis	10 (21.3)	4 (9.1)	0.148
Catheter-associated urinary tract infection (CAUTI)	2 (4.3)	3 (6.8)	0.670
Intra-abdominal infection/gastroenteritis	4 (8.5)	3 (6.8)	0.476
Biliary tract infection	3 (6.4)	8 (18.2)	0.112
Multi-drug resistant infection	2 (4.3)	2 (4.5)	1.00
Fever of unknown origin	2 (4.3)	1 (2.3)	1.00
Most common isolated microorganisms, *n* (%)			
*Escherichia coli*	7 (14.9)	7 (15.9)	1.00
*Klebsiella pneumoniae*	7 (14.9)	3 (6.8)	0.318
Minutes from blood drawing until suPAR result, median (Q1–Q3)	40.0 (35.0–44.0)	40.0 (35.0–45.0)	0.876
Minutes from blood drawing until start of the study drug, median (Q1–Q3)	50.0 (45.0–58.0)	50.0 (47.5–60.0)	0.667
Compliance of antibiotics started after the study drug with the ESCMID guidelines, *n* (%)	40 (85.1)	38 (86.4)	1.00

*ALT* Alanine aminotransferase, *APACHE* acute physiology and chronic health evaluation, *AST* aspartate aminotransferase, *CCI* Charlson’s comorbidity index, *COPD* chronic obstructive pulmonary disease, *ED* emergency department, *ESCMID* European Society of Clinical Microbiology and Infectious Diseases, *MH* medical history, *Q* quartile, *SOFA* Sequential Organ Failure, *n* number of patients, *SD* standard deviation

### Primary endpoint

**Fig. 3:**
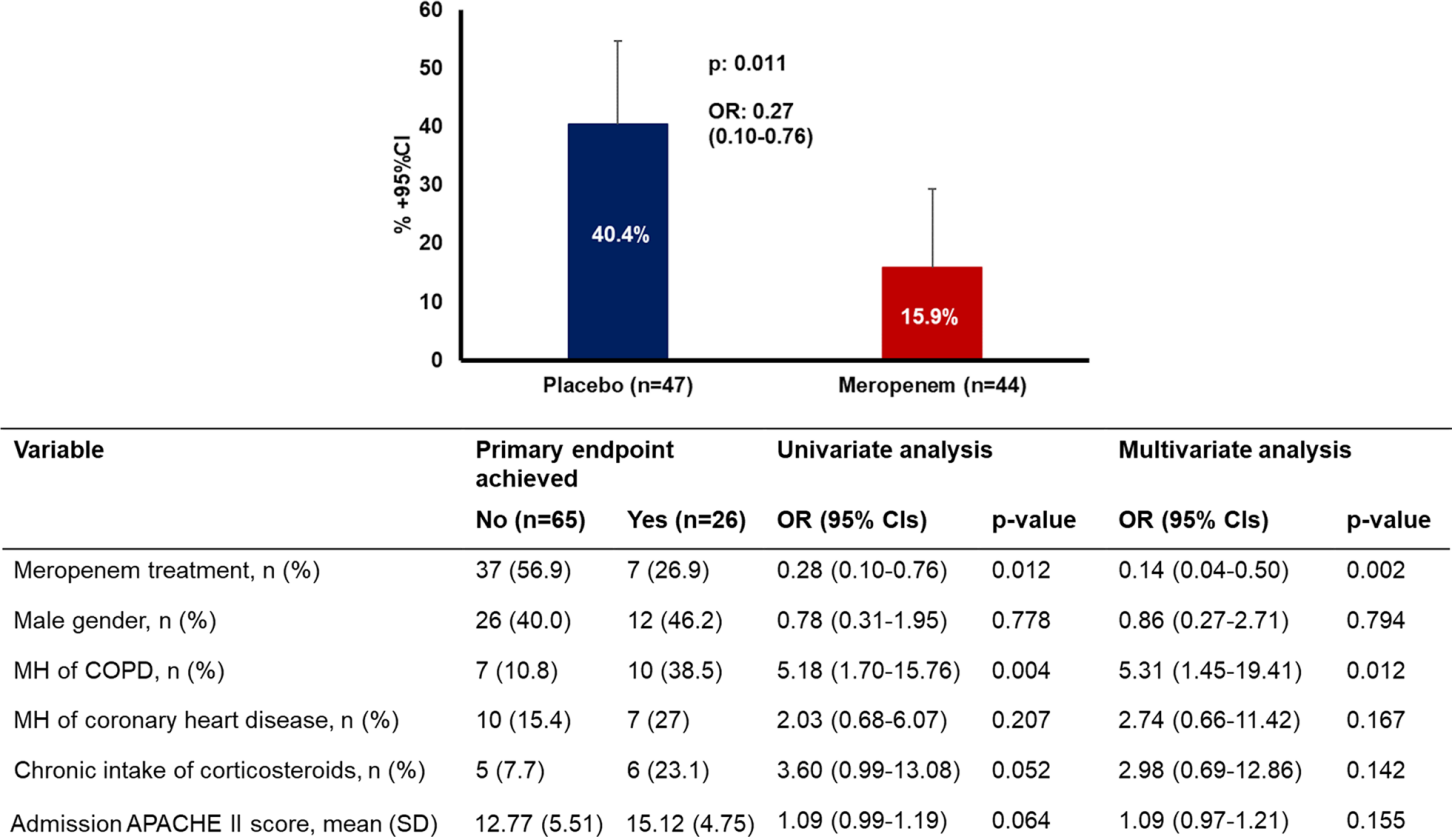
Primary endpoint of the SUPERIOR trial. The graph shows the percent achievement of the primary endpoint in the two groups of treatment. The primary endpoint is early worsening of the patients defined as at least one-point increase of admission SOFA score after 24 h. The table shows the logistic regression analysis of variables associated with primary outcome. Five covariates were included in the multivariate analysis. Three variables (MH of COPD, chronic intake of corticosteroids and admission APACHE II score) had *p* value less than 0.100 in the univariate analysis between achievers and non-achievers of the primary endpoint. Two variables (male gender and MH of coronary heart disease) had *p* value less than 0.100 in the comparisons of the baseline demographics between the two groups of treatment. *APACHE* Acute physiology and chronic health evaluation, *CI* confidence interval, *COPD* chronic obstructive pulmonary disease, *MH* medical history, *n* number of patients, *OR* odds ratio

Early worsening of the patient, defined as at least one-point increase of admission SOFA score, was found in 40.4% (95% CI 26–55%) of patients in the placebo group compared with 15.9% (95% CI 5–27%) in the meropenem group (*p* = 0.011) (Fig. 3A). The relative change of admission SOFA score the first 24 h was higher in the placebo group (*p* = 0.005) (Additional file 5: Fig. S3).

Multivariate logistic regression analysis was performed for the primary endpoint (Fig. 3B). Univariate analysis showed that medical history of chronic obstructive pulmonary disease (COPD), chronic intake of corticosteroids and APACHE II score were the only variables with *p* values of comparisons between achievers and non-achievers of the primary endpoint below 0.100 (Additional file 1: Table S5). Comparisons of the baseline demographics between the two groups showed *p* values less than 0.100 for gender and medical history of coronary heart disease. These five variables were included with the allocation group in the logistic regression model. The model showed that treatment with meropenem was the only variable protecting against early worsening the first 24 h (OR: 0.14, 95% CI 0.04–0.50, *p* = 0.002) and that medical history of COPD was the only variable favoring early worsening.

### Secondary endpoints

Significant differences were found between the two groups of treatment in three secondary endpoints, namely ≥ 2-point increase of admission SOFA score the first 24 h, the rate of infection resolution and the time to infection resolution (Table 2). More precisely, an increase of ≥ 2 points of the admission SOFA score was found in 21.3% (95% CI 9–33%) in the placebo group compared to 4.5% (95% CI 3.2–11%) in the meropenem group (odds ratio 0.17; 95% CI 0.05–0.86, *p* = 0.028). The rate of resolution of infection was 61.7% (95% CI 47–76%) and 84.1% (95% CI 73–95%), respectively, whereas the time to resolution of infection was significantly shorter in the meropenem group.

**Table 2:** Endpoints of the SUPERIOR study

	Placebo (*n* = 47)	Meropenem (*n* = 44)	% Difference (95% CIs)	Odds ratio (95% CI s)	*p* value
Primary endpoint: early worsening, *n* (%)	19 (40.4)	7 (15.9)	24∙5 (5.9 to 40.8)	0.28 (0.10 to 0.76)	0.011
Secondary endpoints					
At least 2-point SOFA increase the first 24 h, *n* (%)	10 (21.3)	2 (4.5)	16∙7 (2.7 to 30.8)	0.18 (0.04 to 0.86)	0.028
Early worsening per quartile of the admission SOFA score, n/patients at the respective quartile (%)					
SOFA = 0-1 points	5/15 (33.3)	0/6 (0)	33.3 (− 0.09 to 0.58)	*	0.262
SOFA = 2–3 points	6/10 (60.0)	7/20 (35.0)	25.0 (− 0.11 to 53.7)		0.255
SOFA = 4 points	5/12 (41.7)	0/8 (0)	41.7 (2.2 to 68.1)	*	0.055
SOFA > 4 points	3/10 (30∙0)	0/10 (0)	30.0 (− 7.7 to 60.3)	*	0.211
Resolution of infection, *n* (%)	29 (61.7)	37 (84.1)	22.4 (4.0 to 38.7)	3∙28 (1.21 to 8.91)	0.020
Time to infection resolution, days, median (Q1-Q3)	13.0 (9 to 60)	12 (8 to 15.8)	NA	NA	0.018
7-day mortality, *n* (%)	2 (4.3)	0 (0)	4.3 (− 4.3 to 14.3)	*	0.494
28-day mortality, *n* (%)	8 (17∙0)	4 (9.1)	7∙9 (− 6.6 to 22.2)	0.49 (0.14 to 1.75)	0.357
60-day mortality, *n* (%)	11 (23.4)	8 (18.2)	5.2 (− 11.7 to 21.5)	0.73 (0.26 to 2.02)	0.611
90-day mortality, *n* (%)	15 (31.9)	9 (20.5)	11.5 (− 6.7 to 28.5)	0.55 (0.21 to 1.43)	0.242
New infections the first 7 days, *n* (%)	4 (8.5)	3 (6.8)	1.7 (− 10.8 to 13.9)	0.79 (0.17 to 3.73)	1.000
New infections the first 60 days, *n* (%)	18 (38.3)	14 (30.2)	6.5 (− 12.8 to 25.0)	0.69 (0.29 to 2.68)	0.507
Change of antibiotics, *n* (%)	24 (51.1)	18 (40.9)	10.2 (− 10 to 29.2)	0.66 (0.29 to 1.52)	0.402
Duration of hospitalization, days, median (range)	8 (5 to 14)	11 (6.3 to 17)	NA	NA	0.372

*Cannot be calculated because one value is zero

*n* Number of patients, *SD* standard deviation, *SOFA* Sequential Organ Failure

Among patients who were not enrolled in the trial, 274 patients failed screening because they had one qSOFA sign but suPAR less than 12 ng/mL; 28-day mortality was 6.6% (95% CI 4.2–7.15). This was significantly lower than the 28-day mortality of patients with one qSOFA sign and suPAR ≥ 12 ng/mL which were allocated to the placebo arm (odds ratio 2.92; 95% CI 1.19–7.16; *p* = 0.036).

### Post hoc analyses

A post hoc analysis revealed that SOFA score after 72 and 96 h decreased significantly more in the meropenem group than the placebo group (Table 3). The empiric treatment administered by physicians as standard of care was modified in 51.1% (95% CI 37.2–64.7%) of patients in the placebo group and 40.9% (95% CI 27.6–55.6%) in the meropenem group [OR = 0.66, 95% CI (0.29–1.52), *p* = 0.402]. De-escalation of the antibiotics initiated after the study drug was done in 34% (95% CI 22.2–48.3%) of placebo patients compared to 22.7% (95% CI 12.8–37%) of patients allocated to meropenem (Table 3). Source control was performed in 8.5% (95% CI 3.4–19.9%) and 13.6% (95% CI 6.4–26.7%) of patients, respectively; 2.1% (95% CI 0.4–11.1) and 2.3% (95% CI 0.4–11.8) required ICU admission (Table 3).

**Table 3:** Post hoc analyses of the SUPERIOR trial

	Placebo (*n* = 47)	Meropenem (*n* = 44)	% difference (95% CIs)	Odds ratio (95% CIs)	*p* value
Delta SOFA 72 h without LOCF, median (Q1 to Q3)	0 (− 1 to 1)^a^	− 1 (− 2 to 0)^b^	NA	NA	0.005
Delta SOFA 96 h without LOCF, median (Q1 to Q3)	0 (− 1 to 0)^c^	− 1 (− 2 to 0)^d^	NA	NA	0.033
Modified empiric treatment after culture results, *n* (%)	24 (51.1)	18 (40.9)	10.2 (− 10 to 29.2)	0.66 (0.29 to 1.52)	0.402
Escalation, *n* (%)	8 (17)	8 (18.2)	1.1 (− 14.6 to 17.2)	1.08 (0.37 to 3.2)	1.00
De-escalation, *n* (%)	16 (34)	10 (22.7)	11.3 (− 7.3 to 28.7)	0.57 (0.23 to 1.44)	0.255
No change, *n* (%)	23 (48.9)	26 (59.1)	10.2 (− 10 to 29.2)	1.51 (0.66 to 3.5)	0.402
Source control performed, *n* (%)	4 (8.5)	6 (13.6)	5.1 (− 8.4 to 19.2)	1.70 (0.45 to 6.5)	0.514
Renal abscess drainage, *n* (%)	2 (4.3)	1 (2.3)	2 (− 8 to 12.2)	0.52 (0.05 to 6)	1.00
Pleural effusion/ empyema drainage, *n* (%)	2 (4.3)	1 (2.3)	2 (− 8 to 12.2)	0.52 (0.05 to 6)	1.00
Pigtail removal, *n* (%)	0	1 (2.3)	2.3 (− 5.5 to 11.8)	0.5 (0.4 to 0.6)	0.484
Percutaneous transhepatic drainage, *n* (%)	0	1 (2.3)	2.3 (− 5.5 to 11.8)	0.5 (0.4 to 0.6)	0.484
Gangrene toe amputation, *n* (%)	0	1 (2.3)	2.3 (− 5.5 to 11.8)	0.5 (0.4 to 0.6)	0.484
Surgery for diverticulitis, *n* (%)	0	1 (2.3)	2.3 (− 5.5 to 11.8)	0.5 (0.4 to 0.6)	0.484
Need for ICU hospitalization, *n* (%)	1 (2.1)	1 (2.3)	0.15 (− 9 to 10)	1.05 (0.1 to 17.3)	1.00

*n* Number of patients, *LOCF* last observation carried forward, *ICU* intensive care unit, *Q* quartile

^a^The total number of patients in the placebo group without LOCF was 44

^b^The total number of patients in the meropenem group without LOCF was 41

^c^The total number of patients in the placebo group without LOCF was 42

^d^The total number of patients in the meropenem group without LOCF was 41

### Safety

No differences in the incidence of serious and non-serious TEAEs were found between the two groups of treatment (Table 4 and Additional file 6: Table S6). Nil TEAE was related to the study drug.

**Table 4:** Most common (> 5%) serious and non-serious treatment-emergent adverse events (TEAEs)

	Placebo (*n* = 47)	Meropenem (*n* = 44)	*p* value
At least one serious TEAE, *n* (%)	24 (51.1)	27 (61.4)	0.399
Type of serious TEAEs, *n* (%)			
Severe anemia and blood transfusion	4 (8.7)	4 (9.1)	1.00
Hemoptysis	0	3 (6.8)	0.113
New hospitalization	7 (15.2)	9 (20.5)	0.588
At least one non-serious TEAE, *n* (%)	44 (93.6)	37 (84.1)	0.188
Type of non-serious TEAEs, *n* (%)			
Diarrhea	7 (14.9)	5 (11.4)	0.605
Constipation	2 (4.3)	4 (9.1)	0.425
Mild anemia	4 (8.7)	6 (13.6)	0.518
Thrombocytopenia	5 (10.9)	5 (11.4)	1.00
Eosinophilia	3 (6.5)	1 (2.3)	0.617
Increased creatinine	4 (8.7)	6 (13.6)	0.518
Increased liver enzymes	9 (19.6)	11 (25.6)	0.613
Blood coagulation disorders	3 (6.5)	3 (6.8)	1.00
Dyspnea	3 (6.5)	2 (4.5)	1.00
Rash	6 (13)	1 (2.3)	0.111
Peripheral edema	3 (6.5)	1 (2.3)	0.617
Hyperglycemia	4 (8.7)	1 (2.3)	0.361
Hypoglycemia	2 (4.3)	5 (11.4)	0.257
Hyponatremia	5 (10.9)	6 (13.6)	0.755
Hypokalemia	11 (23.9)	9 (20.5)	0.802
Hypophosphatemia	3 (6.5)	4 (9.1)	0.711
Hypomagnesemia	2 (4.3)	3 (6.8)	0.673

*n* Number of patients

## Discussion

This study following a two-stage process showed that combining qSOFA score with suPAR at the ED may guide early administration of meropenem and prevent early deterioration of the patient. Even if this trial ended prematurely due to the changes in ED functions during the pandemic, SUPERIOR managed to be successful in achieving the primary endpoint. In addition, four other main endpoints were met; ≥ 2-point increase of admission SOFA the first 24 h was prevented, the resolution of infection was increased and the time to infection resolution was shortened; and finally, the prognostic performance of the qSOFA/suPAR combination was validated. Following a post hoc analysis, the benefit from meropenem treatment on decrease of the SOFA score after 72 and 96 h was also shown.

Although the new Sepsis-3 definitions have reduced the rate of misclassification of critically ill patients, sepsis is still misdiagnosed or diagnosed late in the ED. The role of commonly used rapid assessment tools outside the ICU for the detection of sepsis or septic shock is controversial [8], even in the most recent Surviving Sepsis Campaign 2021 guidelines, in which the single use of qSOFA score is not recommended [13]. Based on two previous validation analyses of the new Sepsis-3 definitions, the sensitivity of qSOFA score ≥ 2 to predict 28-day hospital mortality is close to 60% [5, 7]. This means that a substantial risk for death exists among patients with one point of qSOFA. The SUPERIOR trial showed how risk prediction in these patients may be improved with the use of suPAR guiding early intervention.

Before the publication of the Sepsis-3 criteria, two large studies on the diagnostic and prognostic value of suPAR in sepsis were conducted in Greece. The first included 180 patients hospitalized in two ICUs with sepsis after ventilator-associated pneumonia and showed that suPAR more than 11.9 ng/mL was an independent predictor of unfavorable outcome [9]. The second included 1,914 patients and showed how the combination of suPAR and APACHE (acute physiology and chronic health evaluation) II score improves risk prediction [16].

This is not the first study proving that the addition of suPAR to other existing clinical scores may improve risk detection at the ED. In the TRIAGE III intervention study from Denmark, suPAR prioritized patients in the ED better than the conventional triage algorithm and improved the prediction of short-term mortality risk [17]. The combination to suPAR to the National Early Warning Score (NEWS) [18] is another example. Therefore, the ability to combine suPAR and other biomarkers with vital-sign-based assessment tools is of great importance as there are patients with no or few clinical signs requiring higher attention [9, 17–22]. However, none of these studies proved exactly how the information of early risk detection can guide successful treatment intervention as happened in the double-blind randomized approach of the SUPERIOR RCT.

The present study follows the paradigm generated by the SAVE and SAVE-MORE trials in COVID-19 how the use of suPAR at the ED predicts risk for deterioration and guides early treatment [23, 24]. More precisely, suPAR 6 ng/mL or more increased the likelihood for progression into severe respiratory failure in patients with COVID-19 pneumonia [25]. This risk is substantially attenuated when anakinra, one inhibitor of the interleukin-1 activation, is administered for 10 days guided by the increase of suPAR [23, 24]. During the COVID-19 pandemic, suPAR was proved to predict early deterioration associated with respiratory failure [25] and acute kidney injury [26]. The results of the SUPERIOR trial suggest that for bacterial infections the alert cutoff level of suPAR, prompting intervention, should be 12 ng/mL which is higher than the respective cutoff suggested for viral infections.

SUPERIOR is providing a prospective validation of the qSOFA/suPAR prediction tool generated by the prospective cohort of HSSG. Four main limitations of the SUPERIOR trial need to be mentioned: (a) the small number of study participants due to the premature termination of the study; (b) the limited number of hypotensive patients. Indeed, almost 85% of enrolled patients were tachypneic and few were hypotensive. As such, results cannot be generalized to patients with hypotension where suspicion of infection should guide prompt start of antibiotics [13]; (c) the high prevalence of respiratory tract infections. Indeed, almost half of study participants were sufferers of respiratory tract infections while abdominal tract infections and urinary tract infections were less frequent (25 and 15%, respectively); and (d) the broad-spectrum activity of meropenem. During study design, meropenem was selected due to the high prevalence of infections in the Greek community caused by Gram-negative pathogens producing extended spectrum β-lactamases [27]. The results of the SUPERIOR trial should actually be conceived as the beneficial response after early antibiotic treatment guided by qSOFA/suPAR. With this point of view, the exact type of administered antibiotic may be selected by the results of local surveillance of resistance rates. This may further give rise to the development of larger, international multicenter studies that could corroborate our results.

## Conclusions

Sepsis is a deadly disease and several times early diagnosis escapes. The measurement of the biomarker suPAR in patients with one point of qSOFA score admitted in the ED elaborates risk for unfavorable outcome and early deterioration. These patients receive significant benefit from early meropenem treatment.

### Supplementary Information

**Additional file 1: Table S1.** List of participating sites in the prospective registry. **Table S2.** Baseline characteristics of 2,377 patients of the HSSG cohort study. **Table S3:** Survival analysis of patients enrolled in the prospective cohort study stratified into strata of severity by qSOFA score and serum suPAR. **Table S4.** Antibiotics administered after the study drug. **Table S5.** Comparison of baseline demographics before randomization according to the achievement of the SUPERIOR primary endpoint or not.

**Additional file 2.** Inclusion and exclusion criteria, Methods, and Study design for the prospective registry.

**Additional file 3: Figure S1.** Flowchart of the prospective cohort study. *HSSG* Hellenic Sepsis Study Group, *ICU* intensive care unit, *qSOFA* Quick Sequential Organ Failure Assessment Score, *n* number of patients, *suPAR* soluble urokinase plasminogen activator receptor.

**Additional file 4: Figure S2.** Development of the cutoff of 12ng/mL of suPAR for risk prediction among patients with qSOFA= 1. **A** Receiver operator characteristics curve of suPAR to predict 28-day mortality among patients outside the ICU with qSOFA equal to one. **B** Prognostic performance of suPAR 12ng/mL or more to predict 28-day mortality. *AUC* Area under the curve, *ICU* intensive care unit, *NPV* negative predictive value, *PPV* positive predictive value, *qSOFA* Quick Sequential Organ Failure Assessment Score, *suPAR* soluble urokinase plasminogen activator receptor.

**Additional file 5: Figure S3.** SOFA changes the first 24 h. *n* Number of patients, *SE* standard error.

**Additional file 6: Table S6.** Full list of serious and non-serious treatment-emergent adverse events (TEAEs) classified by system organ class and preferred term.

## Data Availability

After publication, data will be made available to other investigators on reasonable requests to the corresponding author.

## Abbreviations

APACHE: Acute physiology and chronic health evaluation
CI: Confidence intervals
COVID-19: Coronavirus disease 2019
COPD: Chronic obstructive pulmonary disease
DNR: Do not resuscitate
ED: Emergency department
HR: Hazard ratio
HSSG: Hellenic Sepsis Study Group
ICU: Intensive care unit
ITT: Intention-to-treat
IQR: Interquartile range
NEWS: National Early Warning Score
OR: Odds ratio
RCT: Randomized controlled trial
SD: Standard deviation
SIRS: Systemic inflammatory response syndrome
SOFA: Sequential Organ Failure Assessment
qSOFA: Quick Sequential Organ Failure Assessment
suPAR: Soluble urokinase plasminogen activator receptor
TEAEs: Treatment-emergent adverse events
