# Supplementary material for: qSOFA combined with suPAR for early risk detection and guidance of antibiotic treatment in the emergency department: a randomized controlled trial
Source: Crit Care. 2024 Feb 6;28:42. doi: 10.1186/s13054-024-04825-2 (PMC10848347; doi:10.1186/s13054-024-04825-2)
Supplement: Supplementary file 1 — Additional file 1: Table S1. List of participating sites in the prospective registry. Table S2. Baseline characteristics of 2,377 patients of the HSSG cohort study. Table S3: Survival analysis of patients enrolled in the prospective cohort study stratified into strata of severity by qSOFA score and serum suPAR. Table S4. Antibiotics administered after the study drug. Table S5. Comparison of baseline demographics before randomization according to the achievement of the SUPERIOR primary endpoint or not. [file 13054_2024_4825_MOESM1_ESM.docx]

**Additional information**

**qSOFA COMBINED WITH suPAR FOR EARLY RISK DETECTION AND GUIDANCE OF ANTIBIOTIC TREATMENT IN THE EMERGENCY DEPARTMENT: A RANDOMISED CONTROLLED TRIAL**

**Supplementary Tables S1-S4**

**Table S1 List of participating sites in the prospective registry**

| **Participating sites** | **List of approvals** |
| --- | --- |
| 2^nd^Department of Surgery, “G. Gennimatas’’ General Hospital, Thessaloniki | 55/27-06-2008 |
| 2^nd^ Department of Urology, ‘’Sismanogleion’’ General Hospital, Athens | 18606/01-10-2008 |
| 4^th^Department of Internal Medicine ‘’ATTIKON’’ University Hospital | 163/05-06-08 |
| Intensive Care UnitKonstantopouleion Athens General Hospital | 209/06-06-2006 |
| Intensive Care Unit“Laikon”AthensGeneral Hospital | 384/03-07-2006 |
| Intensive Care Unit“Korgialeneio-Benakeio” Athens General Hospital | 226/10-06-2008 |
| 2^nd^Department of Internal Medicine “Sismanogleion”AthensGeneral Hospital | 24380/19-12-2008 |
| 1^st^Department Internal Medicine “Thriasio” Eleusis General Hospital | 38694/29-09-2006 |
| Intensive Care Unit“Ippokrateion”AthensGeneral Hospital | 3004/19-02-2008 |
| Intensive Care Unit“Tzaneion” General Hospital, Piraeus | 15/24-01-2014 |
| Intensive Care Unit“ATTIKON” University General Hospital | 7/18-12-2006 |
| Department of Internal Medicine, Chios General Hospital | 6/03-10-2006 |
| 3^rd^Department of Pulmonary Medicine “Sismanogleion” Athens General Hospital | 2426/07-02-2007 |
| Department of Internal Medicine, Argos General Hospital | 136/07-07-2008 |
| Department of Surgery, Hospital Unit of Nafplio | 4/08-08-2007 |
| Department of Internal Medicine, General University Hospital of Patras | 19956/29-07-2008 |
| Intensive Care UnitGeneral Hospital of Ioannina | 587/06-06-2008 |
| 1^st^Department of Internal Medicine “G. Gennimatas”AthensGeneral Hospital | 4187/19-02-2008 |
| 5^th^Department of Internal Medicine “Evangelismos”AthensGeneral Hospital | 125/02-04-2008 |
| Intensive Care Unit “Bodosakeio” General Hospital of Ptolemaida | 1/28-01-2009 |
| Intensive Care UnitGeneral Hospital of Karditsa | 12/16-12-2008 |
| 1^st^ Department of Surgery “Asklipieion” Voula General Hospital | 6/08-01-2008 |
| Intensive Care Unit“Sotiria” Athens General Hospital | 14207/05-06-2009 |
| Department of Internal Medicine General Hospital Lakonias-Sparti | 67/08-09-2009 |
| Department of Clinical Therapeutics, “Alexandra” Athens General Hospital | 3970/26-11-2009 |
| Intensive Care UnitGeneral Hospital of Trikala | 40/04-12-2009 |
| Department of Internal Medicine. Larissa University General Hospital | 157/04-11-2009 |
| Department of Internal Medicine “Saint Dionysios” General Hospital, Zakynthos | 3231/25-05-2010 |
| 1^st^ Department of Critical Care, “Evangelismos’’ Athens General Hospital | 299/17-12-2014 |
| Burn Intensive Care Unit“G. Gennimatas”AthensGeneral Hospital | 8253/31-03-2010 |
| Department of Urology, Lamia General Hospital | 483/30-05-2011 |
| Intensive Care Unit251 Air Force General Hospital, Athens | 1464/21-01-2011 |
| Intensive Care Unit“KAT” General Hospital, Athens | 38/01-02-2010 |
| Intensive Care Unit“Aghios Dimitrios” General Hospital, Thessaloniki | 14377/24-10-2012 |
| Intensive Care UnitGeneral Hospital of Korinthos | 112/14-12-2011 |
| Intensive Care UnitUniversity General Hospital, Alexandroupolis | 15/10-04-2009 |
| 2^nd^ Department of Internal Medicine “Thriasion” Eleusis General Hospital | 38694/29-09-2006 |
| Intensive Care Unit“Aghios Pavlos” General Hospital, Thessaloniki | 31/07-03-2013 |
| Department of Pulmonary Medicine, Corfu General Hospital | 153/06-04-2015 |

| **Table S2. Baseline characteristics of 2**,**377 patients of the HSSG cohort study** | | | | | |
| --- | --- | --- | --- | --- | --- |
|  | **qSOFA=0**  **(n=590)** | **qSOFA=1 and suPAR<12**  **(n=615)** | **qSOFA=1 and suPAR≥12**  **(n=290)** | **qSOFA≥2**  **(n=882)** | **p-value** |
| Male gender, n (%) | 270 (46.9) | 310 (51.2) | 139 (48.8) | 413 (47.7) | 0.449 |
| Age, mean (SD) | 61.7 (21.7) | 66.6 (20.1) | 75.0 (14.6) | 73.3 (16.8) | <0.001 |
| APACHE II, mean (SD) | 9.97 (6.85) | 12.33 (7.84) | 15.95 (6.65) | 18.96 (8.54) | <0.001 |
| CCI, mean (SD) | 3.03 (2.63) | 3.57 (2.53) | 4.85 (2.51) | 4.74 (2.80) | <0.001 |
| SOFA, mean (SD) | 1.52 (1.93) | 2.60 (2.63) | 3.84 (3.11) | 5.70 (3.91) | <0.001 |
| WBC (/mm^3^), mean (SD) | 14885.42  (8630.50) | 14133.72 (6758.53) | 16259.22 (8914.41) | 16849.82  (11516.31) | <0.001 |
| pO_2_/FiO_2_, mean (SD) | 389 (81.98) | 352.42 (116.54) | 333.33 (115.37) | 300.52 (134.04) | <0.001 |
| **Type of infection, n (%)** | |  |  |  |  |
| AP | 242 (41) | 221 (35.9) | 114 (39.3) | 299 (33.9) | 0.684 |
| CAP | 88 (14.9) | 171 (27.8) | 68 (23.4) | 300 (34) | <0.001 |
| IAI | 173 (29.3) | 143 (23.3) | 59 (20.3) | 148 (16.8) | <0.001 |
| BSI | 36 (6.0) | 36 (5.9) | 31 (10.6) | 64 (7.2) | 0.012 |
| HAP | 6 (1.0) | 10 (1.6) | 7 (1.4) | 29 (3.3) | 0.021 |
| Aspiration pneumonia, n (%) | 6 (1) | 8 (1.3) | 3 (1) | 38 (4.3) | <0.001 |
| ABSSSI | 24 (4.1) | 15 (2.4) | 3 (1) | 17 (1.9) | 0.067 |
| CNS infection | 0 | 1 (0.2) | 2 (0.6) | 3 (0.3) | 0.224 |
| **Comorbidities, n (%)** |  |  |  |  |  |
| Type 2 diabetes mellitus | 127 (21.5) | 136 (22.1) | 94 (32.4) | 273 (31) | <0.001 |
| COPD | 57 (9.7) | 65 (10.6) | 31 (10.7) | 102 (11.6) | 0.206 |
| Chronic heart failure | 80 (13.6) | 98 (15.9) | 60 (20.7) | 180 (20.4) | <0.001 |
| Chronic renal failure | 30 (5.1) | 26 (4.2) | 34 (11.8) | 80 (9.1) | <0.001 |
| Coronary heart disease | 33 (5.6) | 59 (9.6) | 43 (14.8) | 107 (12.1) | <0.001 |
| Atrial fibrillation | 31 (5.3) | 62 (10.1) | 37 (12.8) | 97 (11) | <0.001 |
| Stroke | 46 (7.8) | 79 (12.9) | 46 (16) | 175 (19.9) | <0.001 |
| Dementia | 22 (3.8) | 59 (9.6) | 30 (10.4) | 163 (18.5) | <0.001 |
| Solid tumor malignancy | 60 (10.2) | 83 (13.6) | 56 (19.4) | 153 (17.4) | <0.001 |
| Intake of corticosteroids | 16 (2.7) | 26 (4.2) | 7 (2.4) | 48 (5.5) | 0.015 |
| Abbreviations: ABSSSI, acute bacterial skin and skin structure infections; AP, acute pyelonephritis; APACHE, acute physiology and chronic health evaluation; CAP, community acquired pneumonia; CCI, Charlson’s comorbidity index; COPD, chronic obstructive pulmonary disease; CNS, central nervous system; HAP; hospital acquired pneumonia; IAI, intra-abdominal infection; n, number of patients; SOFA, sequential organ failure; SD, standard deviation; VAP, ventilator-associated pneumonia; WBC, white blood cell count. | | | | | |

| **Table S3**: **Survival analysis of patients enrolled in the prospective cohort study stratified into strata of severity by qSOFA score and serum suPAR** | | | |
| --- | --- | --- | --- |
|  | **Hazard ratio** | **95% CI** | **p-value** |
| qSOFA=1 and suPAR<12 | 1 (baseline) | - | - |
| qSOFA=1 and suPAR≥12 | 2.98 | 2.11-3.96 | <0.001 |
| qSOFA=2 and suPAR<12 | 2.24 | 1.64-3.07 | <0.001 |
| qSOFA=2 and suPAR≥12 | 4.94 | 3.69-6.62 | <0.001 |
| qSOFA=3 and suPAR<12 | 4.1 | 2.83-6.04 | <0.001 |
| qSOFA =3 and suPAR≥12 | 9.16 | 6.63-12.66 | <0.001 |
| Comparisons were done by stepwise Cox regression analysis. Abbreviations:CI, confidence interval; qSOFA, quick Sequential Organ Failure Assessment Score; suPAR, soluble urokinase plasminogen activator receptor | | | |

| **Table S4. Antibiotics administered after the study drug** | | | |
| --- | --- | --- | --- |
|  | **Placebo (n=47)** | **Meropenem (n=44)** | **p-value** |
| β-lactamase inhibitors, n (%) | 2 (4.3) | 1 (2.3) | 1.00 |
| Piperacillin/Tazobactam, n (%) | 23 (48.9) | 19 (43.2) | 0.675 |
| 2^nd^generationcephalosporins, n (%) | 0 | 0 | 1.00 |
| 3^rd^generationcephalosporins, n (%) | 11 (23.4) | 14 (31.8) | 0.483 |
| Carbapenems, n (%) | 5 (10.6) | 5 (11.4) | 1.00 |
| Ceftolozane/Tazobactam, n (%) | 1 (2.1) | 1 (2.3) | 1.00 |
| Quinolones, n (%) | 2 (4.3) | 2 (4.5) | 1.00 |
| Glycopeptides, n (%) | 6 (12.8) | 1 (2.3) | 0.112 |
| Daptomycin, n (%) | 2 (4.3) | 1 (2.3) | 1.00 |
| Linezolid, n (%) | 2 (4.3) | 3 (6.8) | 0.670 |
| Colistin, n (%) | 3 (6.4) | 3 (6.8) | 1.00 |
| Macrolides, n (%) | 5 (10.6) | 10 (22.7) | 0.160 |
| Aminoglycosides, n (%) | 9 (19.1) | 12 (27.3) | 0.457 |
| Antivirals, n (%) | 4 (8.5) | 6 (13.6) | 0.514 |
| Abbreviation: n, number of patients | | | |

**Table S5. Comparison of baseline demographics before randomization according to the achievement of the SUPERIOR primary endpoint or not.**

|  | **Achievement of the primary endpoint** | | **p-value*** |
| --- | --- | --- | --- |
|  | **No (n= 65)** | **Yes (n=26)** |  |
| Male gender, n (%) | 26 (40.0) | 14 (53.8) | 0.642 |
| Age, years, mean (SD) | 72.3 (16.8) | 76.3 (10.8) | 0.278 |
| CCI, mean (SD) | 5.60 (3.21) | 6.12 (2.16) | 0.453 |
| Comorbidities, n (%) |  |  |  |
| Type 2 diabetes mellitus | 19 (29.2) | 10 (38.5) | 0.458 |
| COPD | 7 (10.8) | 10 (38.5) | **0.004** |
| Chronic heart failure | 17 (26.2) | 7 (26.9) | 1.00 |
| Chronic renal disease | 21 (32.3) | 11 (42.3) | 0.469 |
| Coronary heart disease | 10 (15.4) | 7 (26.9) | 0.467 |
| Atrial fibrillation | 18 (27.7) | 9 (34.6) | 0.613 |
| Parkinson’s disease | 3 (4.6) | 1 (3.8) |  |
| Ischemic stroke | 5 (7.7) | 4 (15.4) | 0.269 |
| Chronic intake of corticosteroids | 5 (7.7) | 6 (23.1) | **0.052** |
| Baseline MH SOFA score, mean (SD) | 1.40 (1.62) | 1.50 (1.44) | 0.785 |
| Admission ED SOFA score, mean (SD) | 3.40 (2.29) | 2.81 (1.69) | 0.236 |
| Admission APACHE II score, mean (SD) | 12.77 (5.51) | 15.12 (4.75) | **0.064** |
| White blood cell count, (/mm^3^), mean (SD) | 11897.1 (7033.1) | 9831.3 (3176.6) | 0.158 |
| Platelet cell count, (/mm^3^), mean (SD) | 232062.5 (97018.6) | 268692.3 (122084.2) | 0.136 |
| qSOFA signs, n (%) |  |  |  |
| Respiratory rate ≥ 22/min | 57 (87.7) | 22 (84.6) | 0.737 |
| Systolic blood pressure <100mmHg | 2 (3.1) | 1 (3.8) | 1.00 |
| Altered mental status | 6 (9.2) | 3 (11.5) | 0.711 |
| Minutes from blood drawing until suPAR result, median (Q1-Q3) | 40.0 (35.0-45.0) | 40.0 (35.0-45.0) | 0.874 |
| Minutes from blood drawing until start of the study drug, median (Q1-Q3) | 50.0 (45.0-56.5) | 50.0 (49.3-61.0) | 0.294 |
| Compliance of antibiotics started after the study drug with the ESCMID guidelines, n (%) | 54 (83.1) | 24 (92.3) | 0.335 |

*variables with p-value less than 0.100 are shown in bold

Abbreviations: APACHE, acute physiology, and chronic health evaluation; CCI, Charlson’s comorbidity index; COPD, chronic obstructive pulmonary disease; ED, emergency department; ESCMID, European Society of Clinical Microbiology and Infectious Diseases; MH, medical history; Q: quartile; SOFA, sequential organ failure; n, number of patients; SD, standard deviation.
