# Supplementary material for: qSOFA combined with suPAR for early risk detection and guidance of antibiotic treatment in the emergency department: a randomized controlled trial
Source: Crit Care. 2024 Feb 6;28:42. doi: 10.1186/s13054-024-04825-2 (PMC10848347; doi:10.1186/s13054-024-04825-2)
Supplement: Supplementary file 2 — Additional file 2. Inclusion and exclusion criteria, Methods, and Study design for the prospective registry. [file 13054_2024_4825_MOESM2_ESM.docx]

**Additional information**

**qSOFA COMBINED WITH suPAR FOR EARLY RISK DETECTION AND GUIDANCE OF ANTIBIOTIC TREATMENT IN THE EMERGENCY DEPARTMENT: A RANDOMISED CONTROLLED TRIAL**

**Inclusion and exclusion criteria, Methods, and Study design for the prospective registry**

**Methodology of the HSSG prospective registry**

**Inclusion criteria**

(1) Age ≥ 18 years; male or female;

(2) Time onset of signs of infection within the last 24 hours;

(3) One of the following infections: community acquired pneumonia (CAP), hospital-acquired pneumonia, ventilator-associated pneumonia, acute pyelonephritis, intra-abdominal infection, or primary bacteremia; acute bacterial skin and skin structure infections, central nervous system infections in accordance with internationally accepted definitions;

(4) At least two signs of Systemic Inflammatory Response Syndrome (SIRS).

**Exclusion criteria**

(1) Infection by human immunodeficiency virus (HIV)

(2) Neutropenia, which was defined as less than 1000 neutrophils/mm^3^.

**Methods and study design**

Five thousand and fifty-four patients registered in HSSG biobank from May 2006 until December 2016 and screened for eligibility. The HSSG data included records of all relevant demographic, clinical information, and biological examinations of enrolled participants. Initial sampling was always performed prior to treatment administration. The type of infection and microbiological documentation were reported. Prognostic tools for severity risk such as SOFA, qSOFA, and APACHE II scores and biomarkers of sepsis such as suPAR, CRP, and PCT were also recorded. Only patients outside ICU, admitted in the emergency department for suspected infection, or being already hospitalized in the general ward presenting an infection at least 48 hours after their admission,were analyzed. Patients were followed up until 28 days and the survival status was documented in all cases. Patients were enrolled after written informed consent provided by them or legal representatives.
