# Supplementary material for: qSOFA combined with suPAR for early risk detection and guidance of antibiotic treatment in the emergency department: a randomized controlled trial
Source: Crit Care. 2024 Feb 6;28:42. doi: 10.1186/s13054-024-04825-2 (PMC10848347; doi:10.1186/s13054-024-04825-2)
Supplement: Supplementary file 3 — Additional file 3: Figure S1. Flowchart of the prospective cohort study. HSSG Hellenic Sepsis Study Group, ICU intensive care unit, qSOFA Quick Sequential Organ Failure Assessment Score, n number of patients, suPAR soluble urokinase plasminogen activator receptor. [file 13054_2024_4825_MOESM3_ESM.docx]

**Additional information**

**qSOFA COMBINED WITH suPAR FOR EARLY RISK DETECTION AND GUIDANCE OF ANTIBIOTIC TREATMENT IN THE EMERGENCY DEPARTMENT: A RANDOMISED CONTROLLED TRIAL**

**Supplementary Figure S1**

**
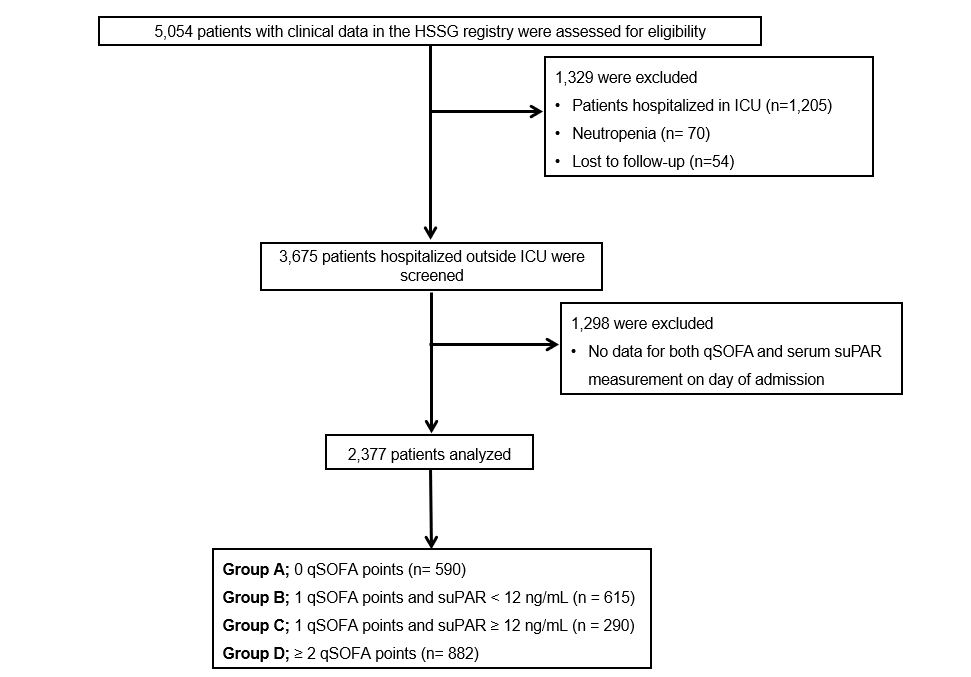
**

**Supplementary Figure1: Flow chart of the prospective cohort study.** Abbreviations:HSSG, Hellenic Sepsis Study Group;ICU, intensive care unit; qSOFA, quick Sequential Organ Failure Assessment Score; n, number of patients; suPAR, soluble urokinase plasminogen activator receptor
