# Supplementary material for: qSOFA combined with suPAR for early risk detection and guidance of antibiotic treatment in the emergency department: a randomized controlled trial
Source: Crit Care. 2024 Feb 6;28:42. doi: 10.1186/s13054-024-04825-2 (PMC10848347; doi:10.1186/s13054-024-04825-2)
Supplement: Supplementary file 4 — Additional file 4: Figure S2. Development of the cutoff of 12ng/mL of suPAR for risk prediction among patients with qSOFA= 1. A Receiver operator characteristics curve of suPAR to predict 28-day mortality among patients outside the ICU with qSOFA equal to one. B Prognostic performance of suPAR 12ng/mL or more to predict 28-day mortality. AUC Area under the curve, ICU intensive care unit, NPV negative predictive value, PPV positive predictive value, qSOFA Quick Sequential Organ Failure Assessment Score, suPAR soluble urokinase plasminogen activator receptor. [file 13054_2024_4825_MOESM4_ESM.docx]

**Additional information**

**qSOFA COMBINED WITH suPAR FOR EARLY RISK DETECTION AND GUIDANCE OF ANTIBIOTIC TREATMENT IN THE EMERGENCY DEPARTMENT: A RANDOMISED CONTROLLED TRIAL**

**Supplementary Figure S2**


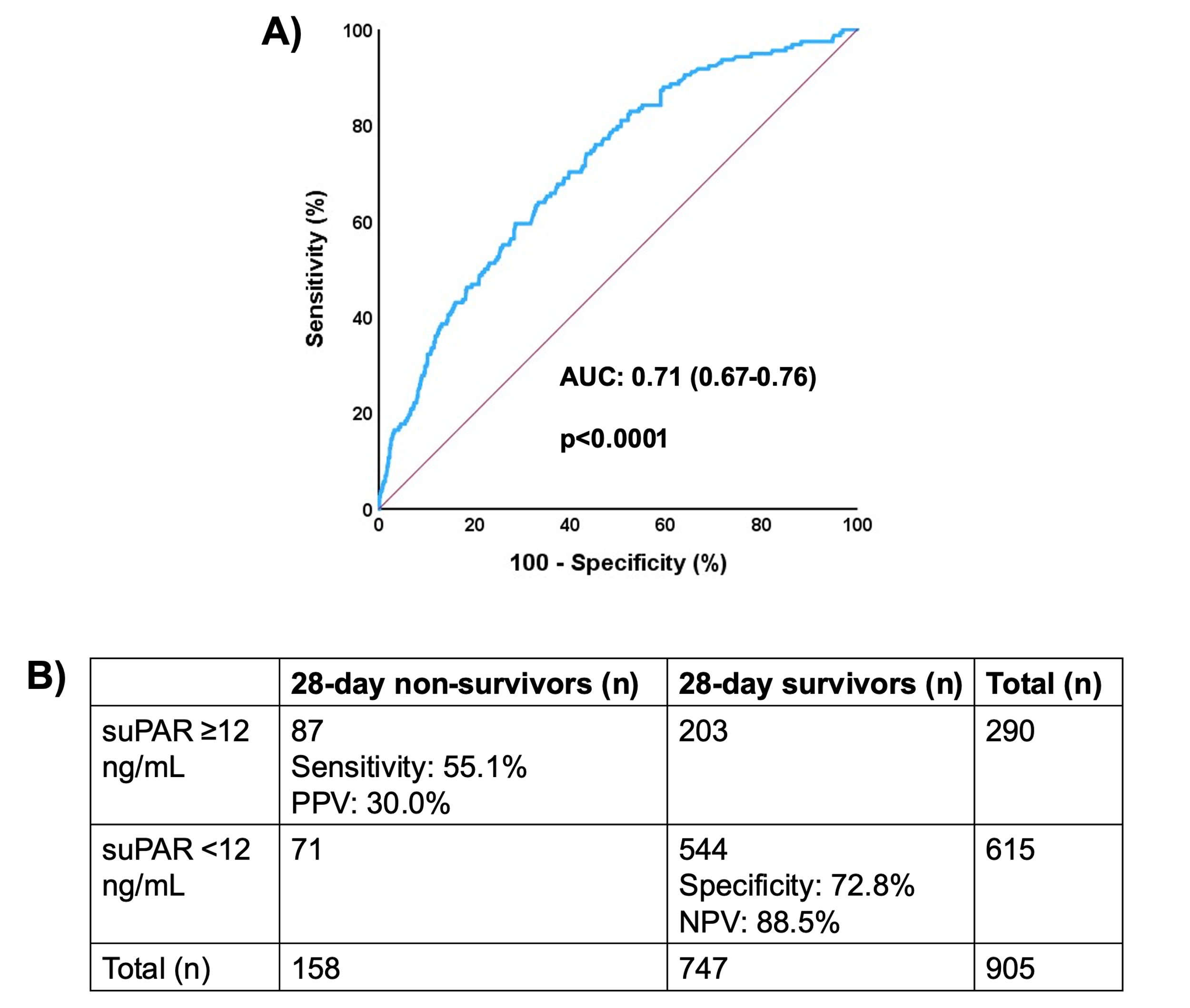


**Supplementary Figure2: Development of the cut-off of 12ng/mL of suPAR for risk prediction among patients with qSOFA= 1.**

A)Receiver operator characteristics curve of suPAR to predict 28-day mortality among patients outside the ICU with qSOFA equal to one.

B)Prognostic performance of suPAR 12ng/mL or more to predict 28-day mortality.

Abbreviations: AUC, area under the curve; ICU, intensive care unit; MPV, negative predictive value; PPV, positive predictive value; qSOFA, quick Sequential Organ Failure Assessment Score; suPAR, soluble urokinase plasminogen activator receptor
