# Supplementary material for: qSOFA combined with suPAR for early risk detection and guidance of antibiotic treatment in the emergency department: a randomized controlled trial
Source: Crit Care. 2024 Feb 6;28:42. doi: 10.1186/s13054-024-04825-2 (PMC10848347; doi:10.1186/s13054-024-04825-2)
Supplement: Supplementary file 5 — Additional file 5: Figure S3. SOFA changes the first 24 h. n Number of patients, SE standard error. [file 13054_2024_4825_MOESM5_ESM.docx]

**Additional information**

**qSOFA COMBINED WITH suPAR FOR EARLY RISK DETECTION AND GUIDANCE OF ANTIBIOTIC TREATMENT IN THE EMERGENCY DEPARTMENT: A RANDOMISED CONTROLLED TRIAL**

**Supplementary Figure S3**


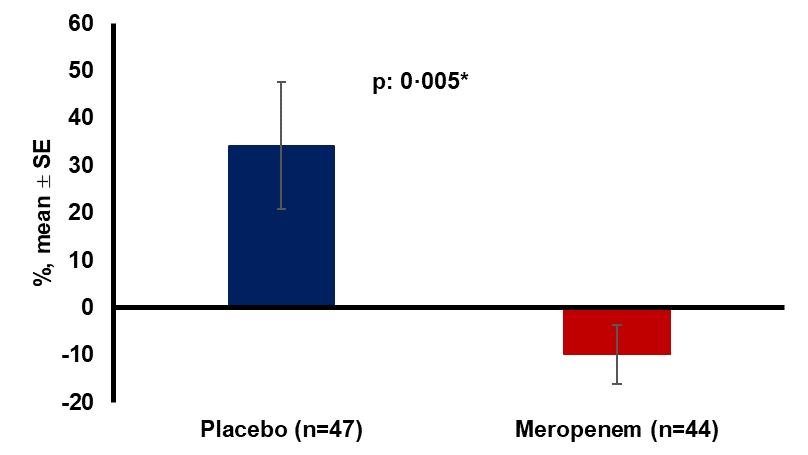


**Supplementary Figure3: SOFA changes the first 24 hours**

Abbreviations: n, number of patients; SE, standard error
