# Supplementary material for: qSOFA combined with suPAR for early risk detection and guidance of antibiotic treatment in the emergency department: a randomized controlled trial
Source: Crit Care. 2024 Feb 6;28:42. doi: 10.1186/s13054-024-04825-2 (PMC10848347; doi:10.1186/s13054-024-04825-2)
Supplement: Supplementary file 6 — Additional file 6: Table S6. Full list of serious and non-serious treatment-emergent adverse events (TEAEs) classified by system organ class and preferred term. [file 13054_2024_4825_MOESM6_ESM.docx]

**Additional information**

**qSOFA COMBINED WITH suPAR FOR EARLY RISK DETECTION AND GUIDANCE OF ANTIBIOTIC TREATMENT IN THE EMERGENCY DEPARTMENT: A RANDOMISED CONTROLLED TRIAL**

**Supplementary Table S6**

**Table S6. Full list of serious and non-serious treatment-emergent adverse events (TEAEs) classified by system-organ class and preferred term**

|  | **SoC + Placebo(n=47)** | **SoC + Meropenem (n=44)** | **Total**  **(n=91)** |
| --- | --- | --- | --- |
| **At least one serious TEAE, n (%)** | 24 (51.1) | 25 (56.8) | 49 (53.8) |
| Probably related | 0 | 0 | 0 |
| Possibly related | 1 (2.1) | 1 (2.3) | 2 (2.2) |
| Possibly not related | 0 | 0 | 0 |
| Not related | 23 (48.9) | 24 (54.5) | 47 (51.6) |
| **Type of serious TEAEs** |  |  |  |
| **Blood and lymphatic system disorders, n (%)** | 4 (8.5) | 4 (9.1) | 8 (8.8) |
| Severe anemia-blood transfusion | 4 (8.5) | 4 (9.1) | 8 (8.8) |
| Probably related | 0 | 0 | 0 |
| Possibly related | 0 | 0 | 0 |
| Possibly not related | 0 | 0 | 0 |
| Not related | 4 (8.5) | 4 (9.1) | 8 (8.8) |
| **Cardiac disorders, n (%)** | 2 (4.3) | 2 (4.5) | 4 (4.4) |
| Atrial fibrillation | 1 (2.1) | 1 (2.3) | 2 (2.2) |
| Probably related | 0 | 0 | 0 |
| Possibly related | 0 | 0 | 0 |
| Possibly not related | 0 | 0 | 0 |
| Not related | 1 (2.1) | 1 (2.3) | 2 (2.2) |
| NSTEMI | 1 (2.1) | 1 (2.3) | 2 (2.2) |
| Probably related | 0 | 0 | 0 |
| Possibly related | 0 | 0 | 0 |
| Possibly not related | 0 | 0 | 0 |
| Not related | 1 (2.1) | 1 (2.3) | 2 (2.2) |
| **Gastrointestinal disorders, n (%)** | 2 (4.3) | 1 (2.3) | 3 (3.3) |
| GI bleeding | 2 (4.3) | 1 (2.3) | 3 (3.3) |
| Probably related | 0 | 0 | 0 |
| Possibly related | 0 | 0 | 0 |
| Possibly not related | 0 | 0 | 0 |
| Not related | 2 (4.3) | 1 (2.3) | 3 (3.3) |
| **General disorders and administration site conditions, n (%)** | 22 (46.8) | 19 (43.2) | 41 (45.1) |
| New fever | 4 (8.5) | 4 (9.1) | 8 (8.8) |
| Probably related | 0 | 0 | 0 |
| Possibly related | 1 (2.1) | 1 (2.3) | 2 (2.2) |
| Possibly not related | 0 | 0 | 0 |
| Not related | 3 (6.4) | 3 (6.8) | 6 (6.6) |
| New hospitalization in 90 days | 7 (14.9) | 9 (20.5) | 16 (17.6) |
| Probably related | 0 | 0 | 0 |
| Possibly related | 0 | 0 | 0 |
| Possibly not related | 0 | 0 | 0 |
| Not related | 7 (14.9) | 9 (20.5) | 16 (17.6) |
| 7-day mortality | 2 (4.3) | 0 | 2 (2.2) |
| Probably related | 0 | 0 | 0 |
| Possibly related | 0 | 0 | 0 |
| Possibly not related | 0 | 0 | 0 |
| Not related | 2 (4.3) | 0 | 2 (2.2) |
| 28-day mortality | 8 (17) | 4 (9.1) | 12 (13.2) |
| Probably related | 0 | 0 | 0 |
| Possibly related | 0 | 0 | 0 |
| Possibly not related | 0 | 0 | 0 |
| Not related | 8 (17) | 4 (9.1) | 12 (13.2) |
| 60-day mortality | 12 (25.5) | 7 (15.9) | 19 (20.9) |
| Probably related | 0 | 0 | 0 |
| Possibly related | 0 | 0 | 0 |
| Possibly not related | 0 | 0 | 0 |
| Not related | 12 (25.5) | 7 (15.9) | 19 (20.9) |
| 90-day mortality | 15 (31.9) | 9 (20.5) | 24 (26.4) |
| Probably related | 0 | 0 | 0 |
| Possibly related | 0 | 0 | 0 |
| Possibly not related | 0 | 0 | 0 |
| Not related | 15 (31.9) | 9 (20.5) | 24 (26.4) |
| **Infections and infestations, n (%)** | 4 (8.5) | 3 (6.8) | 7 (7.7) |
| Empyema | 2 (4.3) | 0 | 2 (2.2) |
| Probably related | 0 | 0 | 0 |
| Possibly related | 0 | 0 | 0 |
| Possibly not related | 0 | 0 | 0 |
| Not related | 2 (4.3) | 0 | 2 (2.2) |
| Liver abscess | 0 | 2 (4.5) | 2 (2.2) |
| Probably related | 0 | 0 | 0 |
| Possibly related | 0 | 0 | 0 |
| Possibly not related | 0 | 0 | 0 |
| Not related | 0 | 2 (4.5) | 2 (2.2) |
| New bacteremia | 1 (2.1) | 1 (2.3) | 2 (2.2) |
| Probably related | 0 | 0 | 0 |
| Possibly related | 0 | 0 | 0 |
| Possibly not related | 0 | 0 | 0 |
| Not related | 1 (2.1) | 1 (2.3) | 2 (2.2) |
| New fungal bloodstream infection | 1 (2.2) | 0 | 1 (1.1) |
| Probably related | 0 | 0 | 0 |
| Possibly related | 0 | 0 | 0 |
| Possibly not related | 0 | 0 | 0 |
| Not related | 1 (2.2) | 0 | 1 (1.1) |
| **Nervous system disorders, n (%)** | 0 | 2 (4.5) | 2 (2.2) |
| TIA/Ischemic attacks | 0 | 2 (4.5) | 2 (2.2) |
| Probably related | 0 | 0 | 0 |
| Possibly related | 0 | 0 | 0 |
| Possibly not related | 0 | 0 | 0 |
| Not related | 0 | 2 (4.5) | 2 (2.2) |
| **Renal and urinary disorders, n (%)** | 1 (2.2) | 3 (6.8) | 4 (4.4) |
| Acute kidney injury | 1 (2.2) | 0 | 1 (1.1) |
| Probably related | 0 | 0 | 0 |
| Possibly related | 0 | 0 | 0 |
| Possibly not related | 0 | 0 | 0 |
| Not related | 1 (2.2) | 0 | 1 (1.1) |
| Oligoanuria | 0 | 3 (6.8) | 3 (3.3) |
| Probably related | 0 | 0 | 0 |
| Possibly related | 0 | 0 | 0 |
| Possibly not related | 0 | 0 | 0 |
| Not related | 0 | 3 (6.8) | 3 (3.3) |
| **Respiratory, thoracic and mediastinal disorders, n (%)** | 2 (4.3) | 4 (9.1) | 6 (6.6) |
| Hemoptysis | 0 | 3 (6.8) | 3 (3.3) |
| Probably related | 0 | 0 | 0 |
| Possibly related | 0 | 0 | 0 |
| Possibly not related | 0 | 0 | 0 |
| Not related | 0 | 3 (6.8) | 3 (3.3) |
| Intubation | 2 (4.3) | 1 (2.3) | 3 (3.3) |
| Probably related | 0 | 0 | 0 |
| Possibly related | 0 | 0 | 0 |
| Possibly not related | 0 | 0 | 0 |
| Not related | 2 (4.3) | 1 (2.3) | 3 (3.3) |
| **At least one non-serious TEAE, n (%)** | 45 (95.7) | 40 (90.9) | 85 (93.4) |
| Probably related | 0 | 0 | 0 |
| Possibly related | 7 (14.9) | 11 (25) | 18 (19.8) |
| Possibly not related | 17 (36.2) | 18 (40.9) | 35 (38.5) |
| Not related | 21 (44.7) | 11 (25) | 33 (36.3) |
| **Type of non-serious TEAEs:** |  |  |  |
| **Blood and lymphatic system disorders, n (%)** | 17 (36.2) | 15 (34.1) | 32 (35.2) |
| Basophilia | 1 (2.1) | 0 | 1 (1.1) |
| Probably related | 0 | 0 | 0 |
| Possibly related | 0 | 0 | 0 |
| Possibly not related | 0 | 0 | 0 |
| Not related | 1 (2.1) | 0 | 1 (1.1) |
| Decreased fibrinogen | 1 (2.1) | 0 | 1 (1.1) |
| Probably related | 0 | 0 | 0 |
| Possibly related | 0 | 0 | 0 |
| Possibly not related | 0 | 0 | 0 |
| Not related | 1 (2.1) | 0 | 1 (1.1) |
| Eosinophilia | 3 (6.4) | 1 (2.3) | 4 (4.4) |
| Probably related | 0 | 0 | 0 |
| Possibly related | 3 (6.4) | 1 (2.3) | 4 (4.4) |
| Possibly not related | 0 | 0 | 0 |
| Not related | 0 | 0 | 0 |
| Increased coagulation time | 3 (6.4) | 3 (6.8) | 6 (6.6) |
| Probably related | 0 | 0 | 0 |
| Possibly related | 0 | 1 (2.3) | 1 (1.1) |
| Possibly not related | 0 | 1 (2.3) | 1 (1.1) |
| Not related | 3 (6.4) | 1 (2.3) | 4 (4.4) |
| Lymphocytosis | 1 (2.1) | 0 | 1 (1.1) |
| Probably related | 0 | 0 | 0 |
| Possibly related | 0 | 0 | 0 |
| Possibly not related | 0 | 0 | 0 |
| Not related | 1 (2.1) | 0 | 1 (1.1) |
| Lymphopenia | 2 (4.3) | 0 | 2 (2.2) |
| Probably related | 0 | 0 | 0 |
| Possibly related | 1 (2.1) | 0 | 1 (1.1) |
| Possibly not related | 1 (2.1) | 0 | 1 (1.1) |
| Not related | 0 | 0 | 0 |
| Mild anemia | 4 (8.5) | 6 (13.6) | 10 (11) |
| Probably related | 0 | 0 | 0 |
| Possibly related | 0 | 0 | 0 |
| Possibly not related | 0 | 4 (9.1) | 4 (4.4) |
| Not related | 4 (8.5) | 2 (4.5) | 6(6.6) |
| Monocytosis | 1 (2.1) | 2 (4.5) | 3 (3.3) |
| Probably related | 0 | 0 | 0 |
| Possibly related | 0 | 0 | 0 |
| Possibly not related | 1 (2.1) | 2 (4.5) | 3 (3.3) |
| Not related | 0 | 0 | 0 |
| Thrombocytosis | 0 | 1 (2.3) | 1 (1.1) |
| Probably related | 0 | 0 | 0 |
| Possibly related | 0 | 0 | 0 |
| Possibly not related | 0 | 1 (2.3) | 1 (1.1) |
| Not related | 0 | 0 | 0 |
| Thrombocytopenia | 5 (10.6) | 5 (11.4) | 10 (11) |
| Probably related | 0 | 0 | 0 |
| Possibly related | 0 | 0 | 0 |
| Possibly not related | 4 (8.5) | 5 (11.4) | 9 (9.9) |
| Not related | 1 (2.1) | 0 | 1 (1.1) |
| **Cardiac disorders, n (%)** | 2 (4.3) | 4 (9.1) | 6 (6.6) |
| Bradycardia | 0 | 1 (2.3) | 1 (1.1) |
| Probably related | 0 | 0 | 0 |
| Possibly related | 0 | 0 | 0 |
| Possibly not related | 0 | 0 | 0 |
| Not related | 0 | 1 (2.3) | 1 (1.1) |
| Tachycardia | 2 (4.3) | 3 (6.8) | 5 (5.5) |
| Probably related | 0 | 0 | 0 |
| Possibly related | 0 | 0 | 0 |
| Possibly not related | 0 | 0 | 0 |
| Not related | 2 (4.3) | 3 (6.8) | 5 (5.5) |
| **Endocrine disorders, n (%)** | 1 (2.1) | 0 | 1 (1.1) |
| Thyroid changes | 1 (2.1) | 0 | 1 (1.1) |
| Probably related | 0 | 0 | 0 |
| Possibly related | 0 | 0 | 0 |
| Possibly not related | 0 | 0 | 0 |
| Not related | 1 (2.1) | 0 | 1 (1.1) |
| **Gastrointestinal disorders, n (%)** | 13 (27.7) | 13 (29.5) | 26 (28.6) |
| Abdominal pain | 2 (4.3) | 3 (6.8) | 5 (5.5) |
| Probably related | 0 | 0 | 0 |
| Possibly related | 0 | 0 | 0 |
| Possibly not related | 0 | 0 | 0 |
| Not related | 2 (4.3) | 3 (6.8) | 5 (5.5) |
| Amylase increased | 2 (4.3) | 0 | 2 (2.2) |
| Probably related | 0 | 0 | 0 |
| Possibly related | 0 | 0 | 0 |
| Possibly not related | 1 (2.1) | 0 | 1 (1.1) |
| Not related | 1 (2.1) | 0 | 1 (1.1) |
| Constipation | 2 (4.3) | 4 (9.1) | 6 (6.6) |
| Probably related | 0 | 0 | 0 |
| Possibly related | 0 | 0 | 0 |
| Possibly not related | 0 | 0 | 0 |
| Not related | 2 (4.3) | 4 (9.1) | 6 (6.6) |
| Diarrhea | 8 (17) | 5 (11.4) | 12 (13.2) |
| Probably related | 0 | 0 | 0 |
| Possibly related | 1 (2.1) | 3 (6.8) | 4 (4.4) |
| Possibly not related | 4 (8.5) | 2 (4.5) | 6 (6.6) |
| Not related | 3 (6.4) | 0 | 3 (3.3) |
| Nausea/Vomiting | 2 (4.3) | 1 (2.3) | 3 (3.3) |
| Probably related | 0 | 0 | 0 |
| Possibly related | 0 | 1 (2.3) | 1 (1.1) |
| Possibly not related | 2 (4.3) | 0 | 2 (2.2) |
| Not related | 0 | 0 | 0 |
| Post-ERCP pancreatitis | 1 (2.1) | 0 | 1 (1.1) |
| Probably related | 0 | 0 | 0 |
| Possibly related | 0 | 0 | 0 |
| Possibly not related | 0 | 0 | 0 |
| Not related | 1 (2.1) | 0 | 1 (1.1) |
| **Generalized disorders and administration site conditions, n (%)** | 4 (8.5) | 2 (4.5) | 6 (6.6) |
| Bedside ulcer | 1 (2.1) | 1 (2.3) | 2 (2.2) |
| Probably related | 0 | 0 | 0 |
| Possibly related | 0 | 0 | 0 |
| Possibly not related | 0 | 0 | 0 |
| Not related | 1 (2.1) | 1 (2.3) | 2 (2.2) |
| Peripheral oedema | 3 (6.4) | 1 (2.3) | 4 (4.4) |
| Probably related | 0 | 0 | 0 |
| Possibly related | 0 | 0 | 0 |
| Possibly not related | 0 | 0 | 0 |
| Not related | 3 (6.4) | 1 (2.3) | 4 (4.4) |
| **Hepatobiliary disorders, n (%)** | 9 (19.6) | 11 (25.6) | 20 (22.2) |
| Increased liver enzymes | 9 (19.6) | 11 (25.6) | 20 (22.2) |
| Probably related | 0 | 0 | 0 |
| Possibly related | 2 (4.3) | 6 (13.6) | 8 (8.8) |
| Possibly not related | 6 (12.8) | 3 (6.8) | 9 (9.9) |
| Not related | 1 (2.1) | 2 (4.5) | 3 (3.3) |
| **Immune system disorders, n (%)** | 6 (13) | 1 (2.3) | 7 (7.7) |
| Rash | 6 (13) | 1 (2.3) | 7 (7.7) |
| Probably related | 0 | 0 | 0 |
| Possibly related | 0 | 0 | 0 |
| Possibly not related | 4 (8.5) | 1 (2.3) | 5 (5.5) |
| Not related | 2 (4.3) | 0 | 2 (2.2) |
| **Infections and infestations, n (%)** | 33 (70.2) | 25 (56.8) | 58 (63.7) |
| ABSSSI | 0 | 1 (2.3) | 1 (1.1) |
| Probably related | 0 | 0 | 0 |
| Possibly related | 0 | 0 | 0 |
| Possibly not related | 0 | 0 | 0 |
| Not related | 0 | 1 (2.3) | 1 (1.1) |
| Cholangitis | 0 | 1 (2.3) | 1 (1.1) |
| Probably related | 0 | 0 | 0 |
| Possibly related | 0 | 0 | 0 |
| Possibly not related | 0 | 0 | 0 |
| Not related | 0 | 1 (2.3) | 1 (1.1) |
| *C. difficile* colitis | 0 | 1 (2.3) | 1 (1.1) |
| Probably related | 0 | 0 | 0 |
| Possibly related | 0 | 0 | 0 |
| Possibly not related | 0 | 1 (2.3) | 1 (1.1) |
| Not related | 0 | 0 | 0 |
| Flu-like symptoms | 1 (2.2) | 0 | 1 (1.1) |
| Probably related | 0 | 0 | 0 |
| Possibly related | 0 | 0 | 0 |
| Possibly not related | 0 | 0 | 0 |
| Not related | 1 (2.2) | 0 | 1 (1.1) |
| LRTI | 17 (36.2) | 10 (22.7) | 27 (29.7) |
| Probably related | 0 | 0 | 0 |
| Possibly related | 0 | 0 | 0 |
| Possibly not related | 0 | 0 | 0 |
| Not related | 17 (36.2) | 10 (22.7) | 27 (29.7) |
| Moniliasis | 0 | 1 (2.3) | 1 (1.1) |
| Probably related | 0 | 0 | 0 |
| Possibly related | 0 | 0 | 0 |
| Possibly not related | 0 | 0 | 0 |
| Not related | 0 | 1 (2.3) | 1 (1.1) |
| New infection in 7 days, n (%) | 4 (8.5) | 3 (6.8) | 7 (7.7) |
| Probably related | 0 | 0 | 0 |
| Possibly related | 0 | 0 | 0 |
| Possibly not related | 0 | 0 | 0 |
| Not related | 4 (8.5) | 3 (6.8) | 7 (7.7) |
| New infection in 60 days, n (%) | 23 (48.9) | 13 (29.5) | 36 (39.6) |
| Probably related | 0 | 0 | 0 |
| Possibly related | 0 | 0 | 0 |
| Possibly not related | 0 | 0 | 0 |
| Not related | 23 (48.9) | 13 (29.5) | 36 (39.6) |
| New infection in 90 days, n (%) | 23 (48.9) | 14 (31.8) | 37 (40.7) |
| Probably related | 0 | 0 | 0 |
| Possibly related | 0 | 0 | 0 |
| Possibly not related | 0 | 0 | 0 |
| Not related | 23 (48.9) | 14 (31.8) | 37 (40.7) |
| Other colitis/gastroenteritis | 0 | 1 (2.3) | 1 (1.1) |
| Probably related | 0 | 0 | 0 |
| Possibly related | 0 | 0 | 0 |
| Possibly not related | 0 | 1 (2.3) | 1 (1.1) |
| Not related | 0 | 0 | 0 |
| SBP | 1 (2.2) | 0 | 1 (1.1) |
| Probably related | 0 | 0 | 0 |
| Possibly related | 0 | 0 | 0 |
| Possibly not related | 0 | 0 | 0 |
| Not related | 1 (2.2) | 0 | 1 (1.1) |
| Sepsis | 8 (17) | 8 (18.2) | 16 (17.6) |
| Probably related | 0 | 0 | 0 |
| Possibly related | 0 | 0 | 0 |
| Possibly not related | 0 | 0 | 0 |
| Not related | 8 (17) | 8 (18.2) | 16 (17.6) |
| Thrombophlebitis | 0 | 1 (2.3) | 1 (1.1) |
| Probably related | 0 | 0 | 0 |
| Possibly related | 0 | 0 | 0 |
| Possibly not related | 0 | 0 | 0 |
| Not related | 0 | 1 (2.3) | 1 (1.1) |
| UTI | 9 (19.6) | 3 (6.8) | 12 (13.2) |
| Probably related | 0 | 0 | 0 |
| Possibly related | 0 | 0 | 0 |
| Possibly not related | 0 | 0 | 0 |
| Not related | 9 (19.6) | 3 (6.8) | 12 (13.2) |
| **Metabolism and nutrition disorders, n (%)** | 21 (44.7) | 25 (56.8) | 46 (50.5) |
| Hyperchloremia | 0 | 1 (2.3) | 1 (1.1) |
| Probably related | 0 | 0 | 0 |
| Possibly related | 0 | 0 | 0 |
| Possibly not related | 0 | 0 | 0 |
| Not related | 0 | 1 (2.3) | 1 (2.1) |
| Hyperglycemia | 4 (8.7) | 1 (2.3) | 5 (5.5) |
| Probably related | 0 | 0 | 0 |
| Possibly related | 0 | 0 | 0 |
| Possibly not related | 0 | 0 | 0 |
| Not related | 4 (8.7) | 1 (2.3) | 5 (5.5) |
| Hyperkalemia | 2 (4.3) | 2 (4.5) | 4 (4.4) |
| Probably related | 0 | 0 | 0 |
| Possibly related | 0 | 0 | 0 |
| Possibly not related | 0 | 0 | 0 |
| Not related | 2 (4.3) | 2 (4.5) | 4 (4.4) |
| Hypermagnesemia | 1 (2.2) | 0 | 1 (1.1) |
| Probably related | 0 | 0 | 0 |
| Possibly related | 0 | 0 | 0 |
| Possibly not related | 0 | 0 | 0 |
| Not related | 1 (2.2) | 0 | 1 (2.1) |
| Hypernatremia | 2 (4.3) | 1 (2.3) | 3 (3.3) |
| Probably related | 0 | 0 | 0 |
| Possibly related | 0 | 0 | 0 |
| Possibly not related | 0 | 0 | 0 |
| Not related | 2 (4.3) | 1 (2.3) | 3 (3.3) |
| Hyperphosphatemia | 0 | 2 (4.5) | 2 (2.2) |
| Probably related | 0 | 0 | 0 |
| Possibly related | 0 | 0 | 0 |
| Possibly not related | 0 | 0 | 0 |
| Not related | 0 | 2 (4.5) | 2 (2.2) |
| Hypochloremia | 0 | 1 (2.3) | 1 (1.1) |
| Probably related | 0 | 0 | 0 |
| Possibly related | 0 | 0 | 0 |
| Possibly not related | 0 | 0 | 0 |
| Not related | 0 | 1 (2.3) | 1 (1.2) |
| Hypoglycemia | 1 (2.1) | 5 (11.4) | 6 (6.6) |
| Probably related | 0 | 0 | 0 |
| Possibly related | 0 | 0 | 0 |
| Possibly not related | 0 | 0 | 0 |
| Not related | 1 (2.1) | 5 (11.4) | 6 (6.6) |
| Hypokalemia | 11 (23.9) | 9 (20.5) | 20 (22) |
| Probably related | 0 | 0 | 0 |
| Possibly related | 0 | 0 | 0 |
| Possibly not related | 0 | 0 | 0 |
| Not related | 11 (23.9) | 9 (20.5) | 20 (22) |
| Hypomagnesemia | 2 (4.3) | 3 (6.8) | 5 (5.5) |
| Probably related | 0 | 0 | 0 |
| Possibly related | 0 | 0 | 0 |
| Possibly not related | 0 | 0 | 0 |
| Not related | 2 (4.3) | 3 (6.8) | 5 (5.5) |
| Hyponatremia | 5 (10.9) | 6 (13.6) | 11 (12.1) |
| Probably related | 0 | 0 | 0 |
| Possibly related | 0 | 0 | 0 |
| Possibly not related | 0 | 0 | 0 |
| Not related | 5 (10.9) | 6 (13.6) | 11 (12.1) |
| Hypophosphatemia | 3 (6.5) | 4 (9.1) | 7 (7.7) |
| Probably related | 0 | 0 | 0 |
| Possibly related | 0 | 0 | 0 |
| Possibly not related | 0 | 0 | 0 |
| Not related | 3 (6.5) | 4 (9.1) | 7 (7.7) |
| **Musculoskeletal and connective tissue disorders, n (%)** | 0 | 1 (2.3) | 1 (1.1) |
| Angiitis | 0 | 1 (2.3) | 1 (1.1) |
| Probably related | 0 | 0 | 0 |
| Possibly related | 0 | 0 | 0 |
| Possibly not related | 0 | 0 | 0 |
| Not related | 0 | 1 (2.3) | 1 (1.1) |
| **Psychiatric disorders, n (%)** | 2 (4.3) | 5 (11.4) | 7 (7.7) |
| Anxiety/Depression | 0 | 1 (2.3) | 1 (1.1) |
| Probably related | 0 | 0 | 0 |
| Possibly related | 0 | 0 | 0 |
| Possibly not related | 0 | 0 | 0 |
| Not related | 0 | 1 (2.3) | 1 (1.1) |
| Delirium | 1 (2.2) | 2 (4.5) | 3 (3.3) |
| Probably related | 0 | 0 | 0 |
| Possibly related | 0 | 0 | 0 |
| Possibly not related | 0 | 0 | 0 |
| Not related | 1 (2.2) | 2 (4.5) | 3 (3.3) |
| Dizziness | 1 (2.2) | 1 (2.3) | 1 (1.1) |
| Probably related | 0 | 0 | 0 |
| Possibly related | 0 | 0 | 0 |
| Possibly not related | 0 | 0 | 0 |
| Not related | 1 (2.2) | 1 (2.3) | 1 (1.1) |
| Insomnia | 0 | 1 (2.3) | 1 (1.1) |
| Probably related | 0 | 0 | 0 |
| Possibly related | 0 | 0 | 0 |
| Possibly not related | 0 | 0 | 0 |
| Not related | 0 | 1 (2.3) | 1 (1.1) |
| **Renal and urinary disorders, n (%)** | 8 (17) | 7 (15.9) | 15 (16.5) |
| Hydronephrosis | 0 | 1 (2.3) | 1 (1.1) |
| Probably related | 0 | 0 | 0 |
| Possibly related | 0 | 0 | 0 |
| Possibly not related | 0 | 0 | 0 |
| Not related | 0 | 1 (2.3) | 1 (1.1) |
| Increased creatinine | 4 (8.5) | 6 (13.6) | 10 (11) |
| Probably related | 0 | 0 | 0 |
| Possibly related | 0 | 1 (2.3) | 1 (1.1) |
| Possibly not related | 3 (6.4) | 5 (11.4) | 8 (8.8) |
| Not related | 1 (2.2) | 0 | 1 (2.1) |
| Hematuria | 1 (2.2) | 1 (2.3) | 2 (2.2) |
| Probably related | 0 | 0 | 0 |
| Possibly related | 0 | 0 | 0 |
| Possibly not related | 0 | 0 | 0 |
| Not related | 1 (2.2) | 1 (2.3) | 2 (2.2) |
| Oliguria | 2 (4.3) | 1 (2.3) | 3 (3.3) |
| Probably related | 0 | 0 | 0 |
| Possibly related | 0 | 0 | 0 |
| Possibly not related | 0 | 0 | 0 |
| Not related | 2 (4.3) | 1 (2.3) | 3 (3.3) |
| Paraphimosis | 1 (2.2) | 0 | 1 (1.1) |
| Probably related | 0 | 0 | 0 |
| Possibly related | 0 | 0 | 0 |
| Possibly not related | 0 | 0 | 0 |
| Not related | 1 (2.2) | 0 | 1 (1.1) |
| **Respiratory, thoracic and mediastinal disorders, n (%)** | 5 (10.6) | 2 (4.5) | 7 (7.7) |
| Dyspnea | 3 (6.4) | 2 (4.5) | 5 (5.5) |
| Probably related | 0 | 0 | 0 |
| Possibly related | 0 | 0 | 0 |
| Possibly not related | 0 | 0 | 0 |
| Not related | 3 (6.4) | 2 (4.5) | 5 (5.5) |
| Nasal epistaxis | 1 (2.2) | 0 | 1 (1.1) |
| Probably related | 0 | 0 | 0 |
| Possibly related | 0 | 0 | 0 |
| Possibly not related | 0 | 0 | 0 |
| Not related | 1 (2.2) | 0 | 1 (1.1) |
| Pneumothorax | 1 (2.2) | 0 | 1 (1.1) |
| Probably related | 0 | 0 | 0 |
| Possibly related | 0 | 0 | 0 |
| Possibly not related | 0 | 0 | 0 |
| Not related | 1 (2.2) | 0 | 1 (1.1) |
